# Pilot, Randomized Study Assessing Safety, Tolerability and Efficacy of Simplified LPV/r Maintenance Therapy in HIV Patients on the 1^st^ PI-Based Regimen

**DOI:** 10.1371/journal.pone.0023726

**Published:** 2011-08-19

**Authors:** Pedro Cahn, Julio Montaner, Patrice Junod, Patricia Patterson, Alejandro Krolewiecki, Jaime Andrade-Villanueva, Isabel Cassetti, Juan Sierra-Madero, Arnaldo David Casiró, Raul Bortolozzi, Sergio Horacio Lupo, Nadia Longo, Emmanouil Rampakakis, Nabil Ackad, John S. Sampalis

**Affiliations:** 1 Fundacion Huesped, Buenos Aires, Argentina; 2 University of British Columbia, Vancouver, Canada; 3 Clinique Médicale du Quartier Latin, Montréal, Canada; 4 Antiguo Hospital Civil de Guadalajara “Fray Antonio Alcalde”, CUCS, Universidad de Guadalajara, Guadalajara, Jalisco, Mexico; 5 Helios Salud, Buenos Aires, Argentina; 6 Instituto Nacional de Ciencias Medicas y Nutricion, Mexico, Mexico; 7 Hospital General de Agudos Teodoro Alvarez, Buenos Aires, Argentina; 8 División Estudios Clínicos, Centro Diagnóstico Médico de Alta Complejidad S.A. (CIBIC), Santa Fé, Argentina; 9 Instituto CAICI, Santa Fé, Argentina; 10 JSS Medical Research, Westmount, Canada; 11 Abbott Laboratories, Montréal, Canada; 12 McGill University, Montréal, Canada; Rush University, United States of America

## Abstract

**Objectives:**

To compare the efficacy and safety of an individualized treatment-simplification strategy consisting of switching from a highly-active anti-retroviral treatment (HAART) with a ritonavir-boosted protease inhibitor (PI/r) and 2 nucleoside reverse-transcriptase inhibitors (NRTIs) to lopinavir/ritonavir (LPV/r) monotherapy, with intensification by 2 NRTIs if necessary, to that of continuing their HAART.

**Methods:**

This is a one-year, randomized, open-label, multi-center study in virologically-suppressed HIV-1-infected adults on their first PI/r-containing treatment, randomized to either LPV/r-monotherapy or continue their current treatment. Treatment efficacy was determined by plasma HIV-1 RNA viral load (VL), time-to-virologic rebound, patient-reported outcomes (PROs) and CD4+T-cell-count changes. Safety was assessed with the incidence of treatment-emergent adverse events (AE).

**Results:**

Forty-one patients were randomized to LPV/r and 39 to continue their HAART. No statistically-significant differences between the two study groups in demographics and baseline characteristics were observed. At day-360, 71(39:LPV/r;32:HAART) patients completed treatment, while 9(2:LPV/r;7:HAART) discontinued. In a Last Observation Carried Forward Intent-to-Treat analysis, 40(98%) patients on LPV/r and 37(95%) on HAART had VL<200copies/mL (P = 0.61). Time-to-virologic rebound, changes in PROs, CD4+ T-cell-count and VL from baseline, also exhibited no statistically-significant between-group differences. Most frequent AEs were diarrhea (19%), headache (18%) and influenza (16%). Four (10%) patients on LPV/r were intensified with 2 NRTIs, all regaining virologic control. Eight serious AEs were reported by 5(2:LPV/r;3:HAART) patients.

**Conclusion:**

At day-360, virologic efficacy and safety of LPV/r appears comparable to that of a PI+2NRTIs HAART. These results suggest that our individualized, simplified maintenance strategy with LPV/r-monotherapy and protocol-mandated NRTI re-introduction upon viral rebound, in virologically-suppressed patients merits further prospective long-term evaluation.

**Trial Registration:**

ClinicalTrials.gov NCT00159224

## Introduction

The standard treatment approach in HIV-1 infection involves using a combination of at least three antiretroviral (ARV) drugs, designated highly active antiretroviral therapy (HAART) to fully suppress plasma HIV-1 RNA viral load (VL), in a sustainable fashion. Currently recommended first line antiretroviral regimens consist of two nucleoside (NRTI) or nucleotide (NtRTI) analog reverse transcriptase inhibitors and either a non-nucleoside reverse transcriptase inhibitor (NNRTI), an integrase strand transfer inhibitor (INSTI) or a ritonavir-boosted protease inhibitor (PI/r) [1]. While adherence to HAART regimens is essential to achieve and maintain long-term virological suppression [2], [3], [4], [5], suboptimal adherence is often observed due to the complexity of the treatment regimens as well as their associated short- and long-term toxicities. The subsequent failure to adequately suppress viral replication permits the rapid selection of resistant mutations, viral rebound and resumption of disease progression [6], [7].

A number of regimen simplification treatment approaches have been explored to improve adherence, reduce the risk of virologic failure and long-term toxicities, and enhance the patient's quality of life [8]. In induction/maintenance therapy, a standard three drug regimen is used to achieve virologic suppression, followed by the use of a simpler regimen to maintain viral control. Lopinavir (LPV) is a PI with potent *in vitro* activity against HIV [9] which has been clinically used in combination with ritonavir (r), a cytochrome P450 3A4 enzyme inhibitor, to enhance its pharmacokinetic properties. LPV/r-based combination ARV regimens have been shown to be effective in the treatment of ARV therapy (ART)-naive patients, both short-term and long-term [4], [10], [11]. However, when used as monotherapy LPV/r was found to achieve lower levels of virologic suppression as compared to LPV/r-based triple ART [12]. In contrast, in a more recent study, Arribas et al. demonstrated that maintenance LPV/r monotherapy was not inferior to triple therapy (LPV/r + 2 NRTIs) in its ability to maintain suppression of the VL among patients with prior stable virologic suppression [13].

The objective of this pilot study was to assess the efficacy and safety of a simplified strategy aimed to optimize the use of LPV/r monotherapy maintenance, whereby intensification with two NRTIs was allowed if VL in plasma became detectable, among patients stably suppressed on PI/r triple-combination therapy.

## Methods

The protocol for this trial and supporting CONSORT checklist are available as supporting information; see Checklist S1 and Protocol S1.

### Patients

Eligible patients were HIV-1 infected adults who: i) were on their first ART regimen, composed of any two NRTIs plus LPV/r or a PI/r combination; and ii) had been virologically suppressed with a HIV-1 RNA viral load of <50 copies/ml for at least 6 months prior to study entry and a CD4+ T-cell count ≥100 cells/mm^3^. Patients were excluded if they were HBsAg+, had active tuberculosis or an opportunistic infection, active malignancy (except Kaposi's Sarcoma), elevated hepatic transaminases (ALT/AST >5x Upper Limit of Normal), or an uncontrolled substance abuse or psychiatric illness that could preclude compliance with the protocol. Patients were also excluded if they were pregnant or lactating, had received an investigational drug within 30 days prior to study initiation, or had modified their ART within three months of study entry or were intending to do so during the course of the study.

### Study Design

This was a one-year pilot, prospective, open-label, randomized, comparative, multi-center study. The study was conducted according to the tenets of the Declaration of Helsinki and approved by an independent ethics review board (Ethica Clinical Research Inc., Montreal, Quebec). All participating patients provided written informed consent prior to study entry. Patients were recruited between January 2005 and July 2007 across 9 sites in Canada, Argentina, and Mexico, and were randomized to receive in a 1∶1 ratio either monotherapy with LPV/r (IM) or the standard HAART regimen (ST). Randomization was centrally coordinated by a third-party data management center and was stratified by center. A sealed envelope containing the randomized allocation was sent by the data management center to the physician who was blinded for the randomization schedule, and was opened by the patient. The allocation document was subsequently signed by the physician and mailed back to the data management center. Patients randomized to the IM group were provided with co-formulated LPV/r 133.3/33.3 mg soft gel capsules and were instructed to take 3 capsules BID orally with food. Clinical assessments took place at Screening/Baseline (Day -1) and Days 15, 30, 60, 90, 120, 150, 180, 240, 300, and 360. Efficacy measures included plasma HIV-1 RNA levels and CD4+ T-cell counts. Safety was assessed with the incidence of treatment emergent adverse events (AE), vital signs, clinical laboratory data, including venous lactic acid and serum lipid levels.

Patients with HIV-1 RNA >50 copies/ml in one visit were retested between 7 and 30 days later. If the second viral load was <50 copies/ml the patients continued on their randomized therapy, while if it was >50 and <200 copies/mL the patients were followed on protocol and were retested until either <50 or >200 copies/ml was confirmed. If the second viral load was >200 copies/ml, patients in the ST arm were considered to have met the endpoint of virologic failure, and treatment was to be modified at the discretion of the investigator/treating physician. In the monotherapy arm, if the second viral load was >200 copies/ml, intensification with two NRTIs was allowed (either the same NRTIs as before randomization or different ones) and the patient was maintained on the randomized treatment starting the visit schedule from the beginning. Intensified patients who developed a viral load of >50 copies/ml and a subsequent viral load of >200 copies/ml were considered to have reached the study endpoint of virologic failure, and therapy was to be modified at the discretion of the investigator or the treating physician.

### Outcome Measures

The primary efficacy endpoint was the percentage of patients with plasma HIV-1 RNA level <200 copies/ml at Day 360. Secondary efficacy measures were the percentage of patients with plasma HIV-1 RNA <50 copies/mL at Day 360 (as determined by the Roche AMPLICOR HIV-1 MONITOR Ultra-Sensitive Assay, version 1.5; lower limit of detection (LLOD)  = 50 copies/mL), the time to confirmed virologic rebound (≥200 copies/ml and ≥50 copies/ml) or meeting the criteria for virologic failure as described above through Day 360, as well as the mean change in Viral Load and CD4+ T-cell count from baseline to final assessment.

The impact on patient-reported outcomes (PROs) was assessed using the Symptoms Distress Module (SDM) which was administered at each visit. This questionnaire was developed by the NIAID AIDS Clinical Trials group and consists of 20 questions evaluating the impact of specific symptoms, possibly related to the treatment, on the patient's life. The total score is calculated as the sum of the five point response to the 20 questions where 0 =  symptom not reported, 1 =  I have this symptom and it doesn't bother me, 2 =  I have this symptom and it bothers me a little, 3 =  I have this symptom and it bothers me, and 4 =  I have this symptom and it bothers me a lot. The SDM score ranges from 0 to 80 and higher values indicate worse PROs.

Safety was determined by the incidence of treatment emergent adverse events (AE), changes in vital signs and clinical laboratory data, as well as the occurrence of metabolic toxicity as indicated by the venous lactic acid and fasting serum lipid levels. AE relationship to the study medication was based on the judgment of the treating physician.

### Statistical Methods

Sample size calculations for the current study were based on the expected difference between the two treatment groups in the proportion of patients with virologic control defined as <200 copies/mL at Day 360. Previous studies have shown that at 48 weeks, approximately 90% of virologically suppressed patients treated with LPV/r remain virologically controlled [14]. In order to detect as statistically significant a relative risk for being virologically suppressed of 1.35 with 80% power and two tailed significance level of 5%, a total of 50 fully evaluable patients per group were required. The study was ended when 80 patients were enrolled due to low recruitment rate.

Between-group differences in the rates of virologic control (proportion of patients with viral load <200 copies/mL and <50 copies/mL at day 360) were assessed with the Chi-Square test. The odds ratio (OR) with 95% confidence intervals was used as the measure of treatment effect. In this analysis the Last Observation Carried Forward (LOCF) approach was used for patients that discontinued the study prior to the 360 day follow up. The time to first confirmed virologic rebound was estimated using the Kaplan Meier Survival function, and the maximum likelihood test was used to compare the two groups with respect to the rate of virologic rebound. The Student's t-test for independent samples was used to assess between group differences with respect to the change in CD4+ T-cell count, VL and SDM score from baseline to final assessment. Repeated Measures Analysis of variance with Mixed Effects to account for unequal follow up were used to assess the treatment effect on CD4+ T-cell count, VL and SDM over time. Paired Student's t-test was used to descriptively assess the change in CD4+ T-cell count, VL and SDM within the two treatment groups, while simple linear regression models were used to assess these changes over time within the two treatment groups. Safety was assessed by the incidence of adverse events. All analyses were performed using the intent-to-treat population, defined as all patients enrolled who had taken at least one dose of the study medications and had completed at least one follow up visit.

## Results

**Figure 1:**
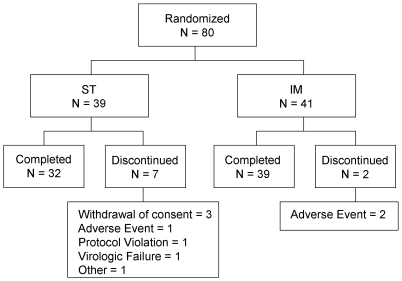
Patient Disposition. * ST: Standard Treatment; IM: Induction/Maintenance.

A total of 80 patients were enrolled in the study, met the intent-to-treat (ITT) criteria and were randomly assigned to treatment, of which, 71 (89%) completed the study and 9 (11%) prematurely discontinued. Among these 9 discontinued patients, 7 belonged to the ST group and 2 to the IM group. Reasons for discontinuation and patient disposition are described in Figure 1.

As summarized in Table 1, demographics and baseline characteristics for the ITT population exhibited no statistically significant differences between groups. The mean (SD) age at screening was 39 (9.3) years. Patients were predominantly males (84%) and Caucasian (94%), with a mean (SD) duration since initial HIV diagnosis of 3.3 (3.0) years. At baseline, the mean (SD) CD4+ T-cell count and log_10_ HIV-1 RNA were 383 (195) cells/mm^3^ and 1.68 (0.08) log_10_copies/ml, respectively. The most common ARV medications used prior to randomization, were: lamivudine 44 (55%), LPV/r 44 (55%), low dose ritonavir 34 (42%), zidovudine/lamivudine 31 (39%), and zidovudine 26 (33%). There were 23 patients on a LPV/r combination and 18 patients on a non-LPV/r regimen in the IM group, prior to randomization. Similarly, 23 patients in the ST group were on a LPV/r combination while 16 patients were on a non-LPV/r combination.

**Table 1:** Patient Demographics and Baseline Characteristics in the ITT population.

Parameter:	ST (N = 39)	IM (N = 41)	Total (N = 80)
**Age (years)**			
Mean (SD)	37.7 (8.51)	39.9 (9.89)	38.9 (9.25)
Median (Range)	37.0 (24.0 – 59.0)	39.0 (23.0 – 75.0)	38.0 (23.0 – 75.0)
**Gender**			
Male; N (%)	36 (92.3%)	31 (75.6%)	67 (83.8%)
**Race: N (%)**			
Caucasian	36 (92.3%)	39 (95.1%)	75 (93.8%)
American Indian/Alaska Native	3 (7.7%)	2 (4.9%)	5 (6.2%)
**Disease duration 2(years)**			
N	26	27	53
Mean (SD)	3.4 (3.85)	3.1 (2.00)	3.3 (3.02)
**Absolute CD4+ T-cell count (cell/mm^3^)**			
N	39	41	80
Mean (SD)	401.2 (222.5)	364.6 (164.3)	382.5 (194.5)
**Viral load (log_10_ RNA copies/mL)**			
N	39	41	80
Mean (SD)	1.689 (0.063)	1.680 (0.087)	1.684 (0.076)

The primary outcome measure of the study was the proportion of patients with plasma HIV-1 RNA <200 copies/ml at 360 days. In an ITT analysis using the LOCF principle, 37 of the 39 patients (95%) in the ST group and 40 of the 41 patients (98%) in the IM group had plasma HIV-1 RNA <200 copies/ml (OR = 0.46; 95% CI: 0.04–5.31; P = 0.611). With respect to the proportion of patients with plasma HIV-1 RNA <50 copies/ml at 360 days, applying again the LOCF principle, there were 36 patients (92%) for the ST and 39 (95%) for the IM group (OR = 0.61; 95% CI: 0.097–3.897; P = 0.671). Four (10%) patients on LPV/r were intensified with 2 NRTIs and all of them regained virologic control, as demonstrated by achieving a plasma HIV-1 RNA <50 copies/mL following the intensification.

**Figure 2:**
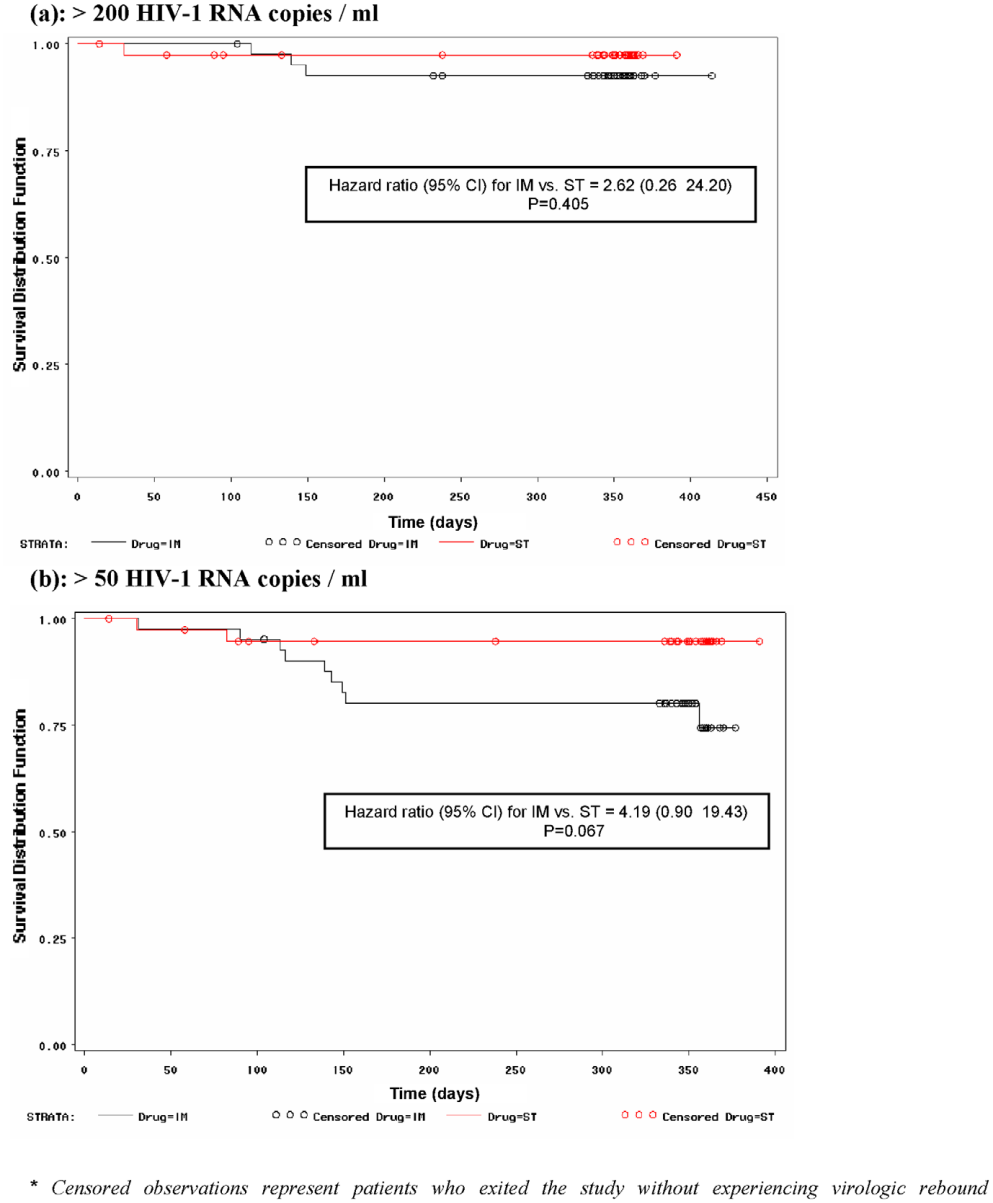
Kaplan Meier Analysis for time to confirmed virologic rebound. (a): >200 HIV-1 RNA copies / ml. (b): >50 HIV-1 RNA copies / ml.* Censored observations represent patients who exited the study without experiencing virologic rebound.

The Kaplan Meier estimates of the proportion of patients with sustained virologic response are shown in Figure 2. Applying the maximum likelihood analysis on these estimates for the time to first confirmed virologic rebound of ≥200 plasma HIV-1 RNA copies/ml, a hazard ratio (95% CI) of 2.62 (0.26–24.20) for IM versus ST was calculated, which was not statistically significant (P = 0.405) (Figure 2a). Similarly, the time to first confirmed virologic rebound of ≥50 HIV-1 RNA copies/ml was comparable in the two groups with an estimated hazard ratio (95% CI) of 4.19 (0.90–19.43), which only approached statistical significance (P = 0.067) (Figure 2b).

The results in Table 2 show that there were no significant between-group differences with respect to the mean changes in CD4+ T-cell counts (P = 0.463) and HIV-1 VL (P = 0.361) from baseline to final assessment. Furthermore, Repeated Measures Analysis of Variance with Mixed Effects indicate that the change in these parameters over time during the 360 day follow up period, was also similar between the two groups (P = 0.794 and P = 0.413, respectively).

**Table 2:** Virologic and Immunologic Response.

Parameter	Visit	ST		IM		Total		P - Value 1
		N	Mean (SD)	N	Mean (SD)	N	Mean (SD)	
Absolute CD4+ T-cell count	Baseline	39	401.2 (222.5)	41	364.6 (164.3)	80	382.5 (194.5)	0.404
	360 days	32	478.6 (246.4)	39	453.8 (249.4)	71	465.0 (246.6)	0.678
	Change	32	56.8 (168.93)	39	89.3 (196.18)	71	74.6 (183.84)	0.463
Viral load log_10_ RNA copies/ml	Baseline	39	1.689 (0.063)	41	1.680 (0.087)	80	1.684 (0.076)	0.592
	360 days	31	1.692 (0.079)	39	1.734 (0.249)	70	1.715 (0.193)	0.369
	Change	31	0.006 (0.032)	39	0.055 (0.245)	70	0.033 (0.184)	0.361

1 *Based on student's t-test for independent samples*.

**Figure 3:**
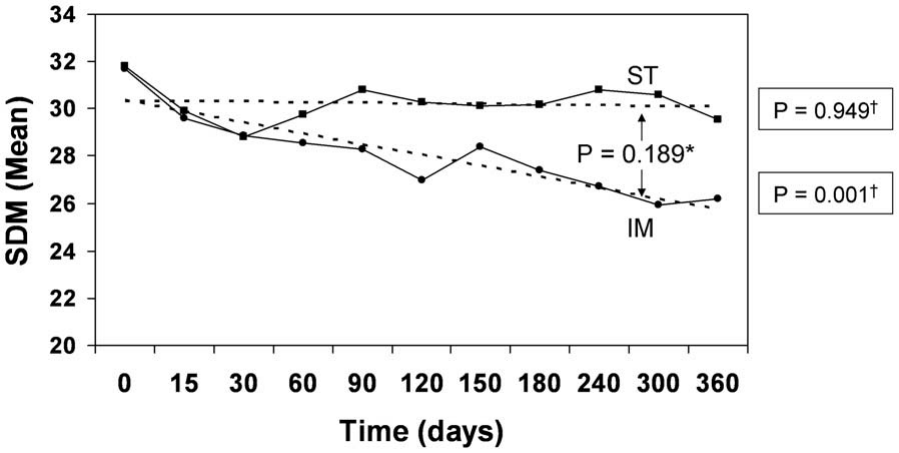
PROs by Treatment Group and Follow-Up Visits. ^1^ Dotted lines represent the linear regression function based on the least squares method. ^2^ SDM, Symptoms Distress Module; ▪, Standard Treatment; ♦, Induction/Maintenance. * P-value for between-group difference in change over time based on Repeated Measures Analysis of Variance ^†^ P-value for within-group change over time based on linear regression analysis.

Using the SDM to assess the PROs, it was determined that the patients in the IM group experienced a decline in the SDM from 31.7 at baseline to 26.2 at 360 days (P = 0.003), indicating a statistically significant improvement in the PROs. On the contrary, patients in the ST group experienced a statistically non-significant decline from 31.8 at baseline to 29.6 at 360 days (P = 0.094). Nevertheless, the difference in the change in SDM from baseline to 360 days of treatment between the two treatment groups was not statistically significant (P = 0.131). Similarly, linear regression analysis showed that the change in SDM over time was statistically significant for the IM group (P = 0.001), but not for the ST group (P = 0.949) (Figure 3). However, Repeated Measures Analysis of Variance again failed to detect a significant between-group difference with respect to the change in SDM over time (P = 0.189).

A total of 658 AEs were reported for 66 (83%) patients. Of these, 269 AEs were reported by 32 (82%) patients in the ST group while 389 AEs were reported by 34 (83%) patients in the IM group. Both the incidence and the profile of adverse events were comparable between the two groups, showing no apparent differences. The most frequently reported adverse events were diarrhea (19%), headache (18%), influenza (16%), nasopharyngitis (13%), back pain (10%), hypertriglyceremia (8%) and insomnia (8%). Adverse events were predominantly mild in severity and judged unrelated to the study drug. There were three SAEs reported by two patients in the IM group (1 thrombocytopenia, 1 upper abdominal pain and 1 pneumonia) and five SAEs reported by three patients in the ST group, of which seven were considered severe and one in the IM group was moderate. All SAEs were considered unrelated to the study drug.

## Discussion

The goal of this pilot, randomized clinical trial was to compare an individualized, simplified maintenance LPV/r-based strategy with reintroduction of two NRTIs upon viral rebound in plasma, to the standard continued triple-drug therapy with respect to sustained virologic response over 360 days, among virologically suppressed HIV-1-infected patients on their first PI/r-based HAART regimen. Our results demonstrate comparable safety, efficacy and tolerability for the induction/maintenance strategy and the continued standard HAART treatment. Overall, virologic success rates of over 90% and 95% were documented when using the 50 and 200 copies/mL plasma viral load thresholds, respectively. Importantly, intensification by NRTIs was required in only 10% of the patients randomized to LPV/r which, in all instances, resulted in regaining virologic control as defined by a sustained plasma HIV-1 RNA level of <50 copies/mL. Additional immunologic and virologic parameters including the change in the CD4-T-cell count and the viral load from baseline to final assessment, the rate of change in these two parameters over the 360 days, and time to virologic rebound defined as >200 HIV-1 RNA copies/ml, were also not statistically different between the two groups. With regards to the time to VL>50 HIV-1 RNA copies/ml, a trend towards the favor of the ST was observed, which was however not statistically significant. Previous studies have shown that ritonavir-boosted PI monotherapy is associated with low-level viremia (50–200 copies/mL), the clinical relevance of which is still not clear [15]. Changes in PROs, as measured by the SDM, favored the induction/maintenance group showing bigger improvement in the patients randomized to the simplified maintenance strategy.

The results of the current study are in agreement with those from the OK04 study [13], [14], showing that 85% vs. 90% and 77% vs. 78% of patients on LPV/r monotherapy vs. patients on standard triple therapy group remained virologically suppressed with HIV-1-RNA levels <50 copies/mL after 48 and 96 weeks, respectively. Furthermore, the longer studies by Pulido et al. [16], Cameron et al. [17] and Nunes et al. [18] also demonstrated comparable virological suppression defined as <50 copies/ml and <80 copies/ml, respectively, between the LPV/r monotherapy and combination therapy arms at 48 months and at later stages.

Use of class-sparing regimens, such as the one described in this study, offers the advantage of saving alternative ARV classes as a “back-up” option for new ARV combinations, in the case of ART failure. Furthermore, such regimens could help avoid the side effects associated with nucleoside analogue-containing regimens including renal or bone toxicity and high cardiovascular risk associated with tenofovir disoproxil fumarate and abacavir, respectively [19], [20], [21]. Mitochondrial toxicity with older NRTIs, currently used in resource-limited settings, was described by Brinkman and coworkers [22], and confirmed by other authors [23], [24]. The clinical presentation of NRTI toxicities seen in HIV-infected individuals is dependent on the organ system affected, including lipoatrophy, lactic acidosis, peripheral neuropathy, hepatic steatosis, myopathy, cardiomyopathy, pancreatitis, bone marrow suppression, lactic acidosis, and the Fanconi syndrome. Hepatic failure with refractory lactic acidosis is the most serious disease complication related to mitochondrial dysfunction [22], [25]. The prescribing information for all NRTIs includes a black-box warning of the potential risk of lactic acidosis, which is constantly updated [26]. Recently, the FDA released a new warning regarding treatment with didanosine (ddI) about a rare, but serious, complication: non-cirrhotic portal hypertension [27]. Stavudine [28], [29] and less frequently zidovudine [30], [31] have also been previously linked to severe lactic acidosis. Although the risk of developing lactic acidosis has fallen due to the dramatic decrease in the number of patients receiving stavudine in the developed world, stavudine continues to be used in developing countries, where cases of severe toxicity continue to be seen [32]. Therefore, the NRTI-sparing strategy might be of particular interest in resource-poor settings whereby, in addition to avoiding the above-mentioned toxicities, more affordable strategies could allow more efficient access to ART.

One of the possible limitations of the current study is the small sample size and the fact that the calculated sample size was not achieved due to low recruitment rate. However, the differences between the two groups with respect to virologic suppression and immunological changes were clinically non-important in addition to not being statistically significant. This observed similarity between the treatment groups provides evidence for the comparability of their effectiveness. Nevertheless, larger studies with longer follow up would be helpful in confirming these conclusions. The open label design of the study represents a methodological limitation. However, this design is in line with real-life practice while the objective and blinded ascertainment of virologic and immunologic parameters precludes the possibility of differential ascertainment bias.

In conclusion, our study reports encouraging preliminary safety and efficacy outcomes using an individualized, simplification maintenance strategy of LPV/r monotherapy with NRTI re-introduction upon viral rebound in plasma, among virologically suppressed patients on their first PI/r-based HAART. Based on these results, our strategy of simplified maintenance with LPV/r monotherapy merits further prospective long term evaluation of its safety and effectiveness in larger cohorts. Evaluation of the simplified strategy proposed here is particularly important as it provides a potentially simple and more affordable strategy for long term ART, that may be particularly relevant to the current global effort to expand access to HAART to millions in need.

## Supporting Information

Protocol S1 (PDF)

Checklist S1 (DOC)
