# Supplementary material for: Pilot, Randomized Study Assessing Safety, Tolerability and Efficacy of Simplified LPV/r Maintenance Therapy in HIV Patients on the 1st PI-Based Regimen
Source: PLoS One. 2011 Aug 19;6(8):e23726. doi: 10.1371/journal.pone.0023726 (PMC3158782; doi:10.1371/journal.pone.0023726)
Supplement: Protocol S1 — (PDF) [file pone.0023726.s001.pdf]

A Pilot, Randomized , Open-Label Study Assessing Safety, Tolerability, Efficacy of a Simplified Lopinavir/Ritonavir-Based Induction/Maintenance Therapy in HIV-Infected Subjects on their First Protease Inhibitor-Based Regimen.

Study Number: ACA-ARGE-04-001

Principal Investigator: Dr Pedro Cahn , MD
Fundación Huésped
Address of Principal Investigator: Pje Peluffo 3932 (1202)
Telephone number of Principal Investigator: +54 11 4981-1855/7777
Fax number of Principal Investigator:+54 11 4982 4024
e-mail address of Principal Investigatorpcahn@huesped.org.ar

Version Number: 8
Date: April 12, 2005 (incorporating amendment #1)

______________________________ ________________
Signature of Principal Investigator Date

1.0 Background Information 4
2.0 Objectives 8
2.1 Primary Objective 8
2.2 Secondary Objectives 8
3.0 Study Design and Procedures 9
3.1 Selection Criteria 10
3.1.1 Inclusion Criteria 10
3.1.2 Exclusion Criteria 11
3.2 Screening Procedures 11
3.3 Study Visits 12
3.3.1 Screening/Baseline (Day –1) 13
3.3.2 Day 15 13
3.3.3 Day 30 13
3.3.4 Day 60 13
3.3.5 Day 90 13
3.3.6 Day 120 14
3.3.7 Day 150 14
3.3.8 Day 180 14
3.3.9 Day 240 14
3.3.10 Day 300 14
3.3.11 Day 360/Termination visit 14
3.4 Study Duration 15
3.5 Discontinuation of Study 16
3.6 Premature Withdrawal of Subjects 16
4.0 Treatments Administered 16
4.1 Prior and Concomitant Therapy 17
4.2 Treatment Compliance 17
5.0 Assessment of Efficacy 17
5.1 Primary Efficacy Variable 17
5.2 Secondary Efficacy Variables 17
6.0 Safety Variables 18
6.1 Adverse Events/Experiences 18
6.1.1 Lipodystrophy 19
6.2 Serious Adverse Events 21
6.3 Toxicity Management 21
6.3.1 Grades 1-2 22
6.3.2 Grades 3-4 22
6.3.3 Hyperglycemia 22
6.3.4 Grade 3 AST/ALT Elevations 23
6.3.5 Pancreatitis/Grade 3–4 Elevations of Pancreatic Amylase 23

6.4 HIV-Related Clinical Events 24
6.5 Subject Management……..24
7.0 Statistics 28
7.1 Justification of Sample Size 28
7.2 Statistical Methods to Address the Study Objectives 28
7.2.1 Efficacy 28
7.2.2 Safety 29
7.3 Level of Significance to be Used 29
7.4 Criteria for Termination of the Study 29
7.5 Procedure for Handling Missing, Unused, and Spurious Data 29
7.6 Procedures for Reporting Deviations from the Original Statistical Plan 29
7.7 Criteria for Selection of Subjects to be Included in the Analyses 29
8.0 Direct Access to Source Data/Documents 30
9.0 Ethical Conduct of the Study 30
9.1 Informed Consent 30
9.2 Conduct of the Study 30
10.0 Data Handling and Record Keeping 31
11.0 Data Quality Assurance 31
12.0 Reference List 32
13.0 Appendices 35
Appendix A. Protocol Synopsis 35
Appendix B. Quality of Life Questionnaire 36
Appendix C. Clinical Toxicity 40
Appendix D. List of Known Expected Manifestations of HIV Infection 46
Appendix E Documents Required Prior to Initiation of the Study 49
Appendix F Responsibilities of the Clinical Investigator 49
Appendix G Elements of Informed Consent 50
Appendix H Participating Investigators 52
Appendix I Optional PK……..54

1.0 Background Information

The standard approach to treating HIV-1 infection in North America involves targeting the viral enzymes, reverse transcriptase and protease, simultaneously, using a cocktail of drugs.[1-3] Such treatment, designated highly active antiretroviral therapy (HAART), normally comprises 3 or 4 agents and includes at least one nucleoside analog reverse transcriptase inhibitor (NRTI) and one or two protease inhibitors (PIs). Although adherence to such a regimen is essential for maintaining suppressive levels of the antiretrovirals, the onerous daily burden of taking so many different pills at different times and under different conditions often leads to poor compliance. The subsequent failure to adequately suppress viral replication permits the rapid selection of resistant mutations, viral rebound and the resumption of disease progression. Furthermore, HAART is associated with short and long-term toxicities including metabolic alterations (insulin resistance, dyslipidemia and mitochondrial toxicities such as lactic acidosis) and morphological changes (peripheral fat wasting and central adiposity).

A variety of new approaches designed to address the problems associated with HAART are currently being explored. These include strategic treatment interruptions to reduce time on therapy, toxicity and cost, the employment of a biphasic, induction/maintenance approach to therapy, where a standard, potent three drug regimen is used to suppress the virus followed by the use of a simpler regimen to maintain viral control while reducing side effects and cost and the use of drug class-sparing regimens to reduce or reverse toxicities such as fat redistribution, dyslipidemia and/or insulin resistance.

This study will assess the safety, tolerability and antiviral activity of a simplified PI-based treatment regimen (Kaletra,™) compared to conventional HAART regimens in patients infected with HIV-1 who are on their first boosted-PI

antiretroviral treatment regimen. Kaletra™ is a co-formulated product that combines 2 PIs, ritonavir and lopinavir, in one dosage form.[4]

One of the problems of HAART addressed by the development of Kaletra™ was poor compliance. The recommended adult dose of ritonavir alone is 600 mg, twice a day for a total of 12 capsules and adherence to this regimen is essential for maintaining optimal viral suppression and preventing the emergence of resistant strains. However, ritonavir is not only a highly selective, competitive, reversible inhibitor of HIV protease, but it is also a potent inhibitor of cytochrome P450 3A4 (CYP3A4), one of the key enzymes involved in the metabolism of PIs. Lopinavir, which also has high specificity for HIV-1 protease, potently inhibits the replication of both wild-type and PI-resistant HIV isolates *in vitro,* but is extensively metabolized by CYP3A4, resulting in low systemic concentrations *in vivo*. Through its action on CYP3A4, co-administered ritonavir strongly inhibits the metabolism of lopinavir and significantly enhances its bioavailability. Indeed, in animal studies, ritonavir was shown to induce plasma lopinavir concentrations that exceed the *in vitro* antiviral EC_{50} in the presence of human serum by >50-fold after 8 h.[5]

Another problem associated with ritonavir used alone was a high incidence of adverse events, particularly in the early stages of treatment, which also contributed to low compliance. However, as CYP3A4 is highly sensitive to inhibition by ritonavir, only low doses are required and the primary pharmacologic activity of the ritonavir component of Kaletra™ is its enhancement of lopinavir pharmacokinetics. Kaletra™ is formulated as capsules containing 133.3 mg lopinavir and 33.3 mg ritonavir, or as a liquid containing 80 mg/mL lopinavir and 20 mg/mL ritonavir and the recommended dose is 3 capsules or 5 ml liquid BID.

The potency of the antiviral activity of Kaletra™ has been clearly demonstrated in a wide spectrum of patients in a number of different clinical trials.[6-9] The durable viral suppression seen after 4 years of therapy[10] proves that it can provide effective, long-term treatment for people with HIV-1. It is active in subjects who have failed other PIs and in subjects with high viral load or low CD_4 count.

Data from one of these trials (M97-720),[6] an ongoing Phase II study of lopinavir/ritonavir in combination with NRTIs suggests there may be a role for monoclass therapy with Kaletra™ in the treatment of HIV-1-infection. In this randomized, double-blind, multicenter study in antiretroviral-naïve subjects, subjects were assigned to receive 200 mg lopinavir/100 mg ritonavir BID or 400 mg lopinavir/100 mg ritonavir BID. One group also received 40 mg stavudine (d4T) BID plus 150 mg lamivudine (3TC) BID starting on Day 1 while the other group did not add d4T and 3TC to their treatment regimen until Day 22. Evaluation of the antiviral activity of lopinavir/ritonavir monotherapy against that of lopinavir/ritonavir + d4T + 3TC after 2 weeks treatment showed that there was a similar decrease in HIV-1 RNA levels in both treatment groups.[6]

Results from an earlier clinical trial (M96 462)[11] had already indicated that PIs administered alone were safe and effective in reducing viral load. Over 80% of subjects in a dose ranging study of ritonavir and saquinavir exhibited an HIV RNA level ≤200 copies/mL after 48 weeks of treatment. Suppression of plasma HIV RNA levels was similar at all dose levels. Investigators were permitted to add NRTI(s) to the ritonavir/saquinavir regimen after week 12 if HIV RNA rose above 200 copies/mL or never declined to <200 copies/mL, but only 25% of subjects on study at Week 48 were receiving NRTI(s) with their PIs. Additional analyses suggested that PI monoclass therapy with ritonavir and saquinavir may result in fewer antiretroviral therapy-associated morphologic abnormalities. Lipoatrophy, the most prevalent body composition abnormality, was more

common among subjects receiving NRTIs compared to subjects on NRTI-sparing regimens[12].

PI monoclass therapy may also induce fewer metabolic abnormalities than conventional HAART. Data from a recent study in which 10 HIV-seronegative men were treated with lopinavir/ritonavir over 4 weeks showed that they did not demonstrate significant changes in insulin-mediated glucose clearance, suggesting that lopinavir/ritonavir administered as a single agent may not increase insulin resistance.[13]

Recently, results from 2 studies examining the safety and efficacy of lopinavir/ritonavir as monoclass therapy in HIV-1-infected subjects have been reported. In the first study,[14] adverse effects attributed to other antiviral medications in a planned Kaletra™ combination regimen led to the use of lopinavir/ritonavir monotherapy. A total of 15 subjects, most of who had advanced disease (CD_4 cell count <350 cells/mm3) and had been previously treated with at least two different PIs, were included in the study. Each subject received at least 8 weeks of monoclass therapy with lopinavir/ritonavir.

The mean CD_4 cell count increased from 171 cells/mm^3 prior to treatment to 298 cells/mm^3 after lopinavir/ritonavir monotherapy. A majority of the subjects (12/15, 80%) had HIV RNA levels <400 copies/mL and 9/12 (75%) had HIV RNA levels < 50 copies/mL at last available follow-up. Five subjects continued on lopinavir/ritonavir monotherapy for more than one year with sustained viral suppression (<50 copies/mL). Only 3 subjects, 1 of whom had documented non-adherence and another whose viral load decreased from 54,600 copies/mL pre-lopinavir/ritonavir to1950 copies/mL at week 12 failed to achieve virologic suppression after 8 weeks on lopinavir/ritonavir monotherapy. Protease gene mutations were noted in 2 subjects following exposure to lopinavir/ritonavir, including the subject with documented non-adherence.

A second, ongoing pilot study,[15] is assessing the safety and efficacy of lopinavir/ritonavir as monotherapy in 30 HIV-1-infected, antiretroviral-naïve subjects. Results at week 24 of treatment suggest that the virologic efficacy of Kaletra™ is comparable to that of triple drug HAART and that monoclass therapy is not associated with significant toxicity or genotypic/phenotypic resistance.

The proposed study will involve 100 HIV-1-infected subjects on their first PI-based antiretroviral therapy and will compare the safety, tolerability and efficacy of monoclass therapy with lopinavir/ritonovir to that of treatment regimens which include 2 NRTIs. The study will be conducted in accordance with good clinical practices and the protocol. All applicable clinical research regulations and guidelines as well as all applicable local regulations will be adhered to.

2.0 Objectives

2.1 Primary Objective

The primary objective of this study is to assess the efficacy, and safety of the strategy of switching to a simplified lopinavir/ritonavir-based treatment regimen with intensification by reinitiation of 2 NRTI's if necessary, compared to the strategy of continuing on a current regimen comprising 2 NRTIs plus a ritonavir-boosted PI in patients infected with HIV-1 who are on their first PI-based anti-retroviral therapy.

2.2 Secondary Objectives

The secondary objectives of the study are to:

.

- Evaluate the resistance profile of the simplified treatment *vs.* the control group.

- Evaluate the comparative cost of both arms at the end of the study period.

3.0 Study Design and Procedures

This is a pilot, prospective, randomized, open-label, comparative, multi-center study in HIV-infected adults on their first PI-based anti-retroviral therapy. Approximately 100 subjects will be enrolled at sites in Canada, Argentina, and Mexico.

At the first visit (Screening/Baseline Visit), subjects will undergo screening procedures as listed in Section 3.2 and will be randomly assigned to receive either lopinavir/ritonavir as monotherapy (study group) or to continue on their current anti-retroviral treatment regimen (control group) for the next 360 days.

The measurements made during the screening period, as described in Section 3.2, will serve as baseline measurements for the study. Subjects will return to the clinic on Day 15 for check-up, assessment of their compliance to the treatment regimen and to report adverse events. Return visits will be scheduled for Day 30 and then every 30 days until Day 180. After day 180, subjects will return for visits at Day 240, 300 and 360. The procedures to be carried out at these visits are summarized in Table 1.
Direct costs only related to the study medication will be captured. If as expected, no significant differences in outcomes/effectiveness are observed, a cost minimization analysis will be performed as per standard methodology. If the QOL estimates show a difference in effectiveness between the treatment regimens, we will additionally perform a cost utility analysis and estimate the incremental cost per life year gained.

The planned duration of the study is 360 days (12 months).

3.1 Selection Criteria

3.1.1 Inclusion Criteria

- Subject has confirmed his or her willingness to participate in this study after being informed of all aspects of the trial that are relevant to his or her decision to take part, by signing and dating the IRB / IEC approved informed consent form.
- Subject is HIV positive and on their first antiretroviral treatment regimen, based on any two NRTIs plus lopinavir/ritonavir or a ritonavir-boosted PI combination. Subject must have had no previous exposure to other regimens.
- Subject has a viral load <50 copies/ml at the time of baseline evaluation for at least 6 months.
- Subject has a CD4 cell count ≥ 100 cells/mm3.
- Subject is aged ≥18 years.
- Vital signs, physical examination and laboratory results do not exhibit evidence of acute illness.
- Subject has not been treated for an active opportunistic infection within 30 days of screening.
- If female, subject has a negative pregnancy test and agrees to use, for the duration of the study, a barrier method of birth control that has a history of proven reliability as judged by the investigator.
- Subject does not require and agrees not to take, for the duration of the study, any medication that is contraindicated with any of the antiretroviral drugs in their treatment regimen. The subject agrees not to take any medication, including over-the-counter

medicine, alcohol, or recreational drugs without the knowledge and permission of the principal investigator.

3.1.2 Exclusion Criteria

- Subject has current uncontrolled substance abuse or psychiatric illness that could preclude compliance with the protocol.
- Subject has a viral load of > 50 copies/ml
- Subject is HBsAg +
- Subject has active tuberculosis or an opportunistic infection.
- Subject has active malignancy (except Kaposi's Sarcoma).
- Subject has liver failure as evidenced by ALT / AST > 5 x Upper Limit of Normal (ULN).
- Female subject is pregnant or lactating.
- Subject has received an investigational drug within 30 days prior to the initiation of the study.
- Subject has modified his/her antiretroviral therapy during the 3 months prior to baseline or is intending to do so during the course of the study.

3.2 Screening Procedures

After the subject signs and dates the study-specific informed consent, he/she will undergo the following screening procedures:

- Complete medical history, including smoking, alcohol and drug history and prior medications.
- Urine or serum pregnancy test for females of reproductive potential.

- Complete physical examination, including height, weight and abdominal circumference measured at the umbilicus level while standing.
- Vital signs including systolic and diastolic blood pressures, pulse, respiration rate and body temperature.
- Plasma HIV RNA level, measured using the Roche Amplicor assay.
- CD_4 cell count.
- Clinical laboratory tests: Complete blood count(CBC); glucose; blood urea nitrogen (BUN); Hepatitis B Surface Antigen (HBsAg);serum creatinine; serum glutamic-pyruvic transaminase (SGPT/ALT); serum glutamic-oxaloacetic transaminase (SGOT/AST); serum amylase; sodium; potassium; fasting serum lipids including TG, total cholesterol, HDL and LDL. A 12 hours fasting period is required. Venous lactic acid.
- Quality of Life (QOL) questionnaire.
- Concomitant medications (Section 4.1).

The results of all clinical evaluations during screening must be within clinically acceptable limits as defined by the test laboratory and reviewed by the investigator. Subjects will not be included in the study if laboratory or other screening results are unacceptable.

3.3 Study Visits

The timing of study visits and the procedures that will be carried out at each visit are summarized in Table 1 – Schedule of Procedures.

3.3.1 Screening/Baseline (Day –1)

Screening procedures as described in Section 3.2 will be performed during a period of two to three weeks before the Baseline Visit (Day-1), the day prior to the initiation of study treatment.

3.3.2 Day 15

- Physical examination including weight and abdominal circumference in the upright position.
- Vital signs, viral load, QOL questionnaire and concomitant medications as for Screening/Baseline Visit.
- Compliance (Section 4.2).
- Adverse events (Section 6.0).

3.3.3 Day 30

- Physical examination as for Day 15
- Vital signs, viral load, CD_4 count, clinical laboratory tests, , QOL questionnaire and concomitant medications as for Screening/Baseline Visit.
- Compliance and adverse events as for Day 15

3.3.4 Day 60

As for Day 15

3.3.5 Day 90

As for Day 30

3.3.6 Day 120

As for Day 30

3.3.7 Day 150

As for Day 30

3.3.8 Day 180

As for Day 30

3.3.9 Day 240

As for Day 30

3.3.10 Day 300

As for Day 30

3.3.11 Day 360/Termination Visit

As for Day 30

Table 1: Schedule of Procedures

Procedures	Day –1/ Screening/ Baseline	Day 15	Day 30	Day 60	Day 90	Day 120	Day 150	Day 180	Day 240	Day 300	Day 360 Termination Visit
Informed Consent	X										
Medical History	X										
Pregnancy Test[5]	X										
Physical Exam[1]	X	X	X	X	X	X	X	X	X	X	X
Vital Signs	X	X	X	X	X	X	X	X	X	X	X
HIV RNA Level	X	X	X	X	X	X	X	X	X	X	X
CD_4 cell count	X		X		X	X	X	X	X	X	X
Other Hematology[2]	X		X		X	X	X	X	X	X	X
Fasting Serum Lipids[3]	X		X		X	X	X	X	X	X	X
Venous Lactic Acid	X		X		X	X	X	X	X	X	X
Other Chemistry[4]	X		X		X	X	X	X	X	X	X
HBsAg	X										
QOL Questionnaire	X	X	X	X	X	X	X	X	X	X	X
Concomitant Medications	X	X	X	X	X	X	X	X	X	X	X
Monitor Compliance		X	X	X	X	X	X	X	X	X	X
Monitor Adverse Events		X	X	X	X	X	X	X	X	X	X

[1] including abdominal circumference
[2] CBC
[3]TG; total cholesterol; HDL; LDL
[4]glucose; BUN; creatinine; SGOT (AST); SGPT (ALT); amylase; sodium; potassium
[5] Urine or serum pregnancy test to be done at Day –1 for women of child-bearing potential, and at other visits if clinically indicated

3.4 Study Duration

It is estimated that the duration of this study will be 12 months. Enrolment of subjects will take 18 months and each subject randomized to receive lopinavir/ritonavir monotherapy will be treated for 360 days.

3.5 Discontinuation of Study

See Section 7.4.

3.6 Premature Withdrawal of Subjects

Each subject has the right to withdraw from the study at any time. The investigator may discontinue any subject's participation if the investigator deems it necessary for any reason, including adverse events, safety concerns, and failure to comply with the protocol. The participation of any female subject who becomes pregnant during the course of the study will be discontinued.

In the case of premature discontinuation from the study, a termination visit will be completed.

Any subject who discontinues study participation with an unresolved clinically significant laboratory abnormality or adverse event will be followed for 30 days after the last dose of lopinavir/ritonavir or until satisfactory clinical resolution is achieved.

4.0 Treatments Administered

Subjects randomized to the monotherapy group will be provided with co-formulated lopinavir/ritonavir 133.3/33.3 mg (Kaletra™) soft gel capsules manufactured by Abbott Laboratories. They will be instructed to take 3 capsules BID orally with food. Subjects randomized to the control group will continue on their current treatment regimen.

4.1 Prior and Concomitant Therapy

Subjects will be provided with a list of medications that should not be taken during the study. They will be advised to avoid taking any medication that is contraindicated.

Any medication (including over-the-counter medicines such as aspirin, antacids, vitamins, mineral supplements and herbal preparations) that the subject is receiving at the time of enrollment, or receives during the study, must be recorded together with the dates of administration and dosages. Any previous antiretroviral therapy should be recorded along with the dates of administration. Any vaccine administered to the subject should be listed as a concurrent medication.

4.2 Treatment Compliance

All subjects will receive ongoing counseling regarding the importance of compliance with their medication. They will be instructed to return the container of study drug, whether empty or containing drug, to the study coordinator at all study visits following the Screening/Baseline Visit.. . A pill count at every visit will be used to assess compliance with treatment.

5.0 Assessment of Efficacy

5.1 Primary Efficacy Variable

The primary efficacy variable will be the percentage of subjects with plasma HIV RNA level < 200 copies/ml at Day 360.

5.2 Secondary Efficacy Variables

The secondary efficacy variables will be:

1. Percentage of subjects with plasma HIV RNA < 50 copies/mL at Day 360.
2. The time to confirmed virologic rebound (>200 and >50) through Day 360.
3. The mean change from baseline to each visit in HIV RNA level and CD_4 cell count.
4. The emergence of resistant strains.

6.0 Safety Variables

Safety will be assessed from adverse events, physical examination, vital signs and clinical laboratory data. In addition, safety will be evaluated by examining:

- Metabolic toxicity by measuring venous lactic acid and fasting serum lipid levels.

6.1 Adverse Events/Experiences

Throughout the course of the study (which begins when the Informed Consent is signed), the investigator will monitor each subject for the development of any clinical and/or laboratory evidence of an Adverse Event/Experience (AE). An adverse event/experience is defined as any undesirable medical occurrence in a subject who participates in a study and includes those events/experiences which do not necessarily have a causal relationship to the study drug regimen. Prior to the administration of study drug, only adverse events/experiences that meet the definition of serious (Section 8.3) and adverse events that the investigator considers to be related to study design and/or procedures should be recorded.

An adverse event may be a symptom, sign, or abnormal laboratory finding. Any worsening of a pre-existing condition or intercurrent illness should be reported as an adverse event/experience. A laboratory abnormality should be reported as an adverse event/experience if action is required (e.g. study drug interruption, discontinuation, or treatment is required). The nature of the adverse sign or symptom, its date and time of onset, duration and severity, therapy employed (if any) and the investigator's opinion of causality to study drug with an alternate etiology, if appropriate, must be documented. For adverse events/experiences to be considered as intermittent or continuous, the events should be of similar nature and severity.

The investigator will follow all adverse events to satisfactory clinical resolution.

The investigator will rate the severity of the adverse event according to the following definition:

Mild:	The adverse event is transient and easily tolerated by the subject.
Moderate:	The adverse event causes the subject discomfort and interrupts the subject's normal activities.
Severe:	The adverse event causes considerable interference with the subject's normal activities, and may be incapacitating or life-threatening.

The investigator will use the following definitions to assess the relationship of the adverse event to study drug:

Probable:	An adverse event has a strong temporal relationship to study drug or recurs on rechallenge, and another etiology is unlikely or significantly less likely.
Possible:	An adverse event has a strong temporal relationship to the study drug, and an alternative etiology is equally or less likely compared to the potential relationship to study drug. The alternate etiology should be provided by the investigator.
Probably Not:	An adverse event has little or no temporal relationship to the study drug and/or a more likely alternative etiology exists. The alternate etiology should be provided by the investigator.
Not Related:	An adverse event is due to underlying or concurrent illness or effect of another drug and is not related to the study drug. The alternate etiology should be provided by the investigator.

6.1.1 Lipodystrophy

Fat redistribution and Cushingoid appearance without Cushing's disease have been reported as effects that have been seen in HIV-infected/AIDS

patients receiving potent antiretroviral therapy including the commercially available protease inhibitors. To date, the mechanisms leading to and the long-term consequences of these effects are unknown. A causal relationship to the protease inhibitors has not been established. Such conditions have been grouped under the heading of “lipodystrophy.” However, at present no consensus definition exists. Therefore, for the purposes of adverse event reporting within this study, the following conditions should be reported as discrete events rather than being clustered under the term “lipodystrophy:”

- Peripheral fat wasting (including face, buttocks, and limbs)
- Central adiposity
- Breast hypertrophy
- Dorsal fat pad (“buffalo hump”)
- Multiple lipomas
- Cushingoid appearance without Cushing’s disease.

Metabolic abnormalities such as hyperlipidemia or hyperglycemia have not been consistently observed with abnormalities of fat distribution and should not be labeled as lipodystrophy for adverse event reporting purposes.

6.2 Serious Adverse Events

The investigators will inform Abbott Laboratories and the Regulatory Agency (if applicable) of any serious adverse event reported in this study.
A Serious Adverse Event (SAE) is an adverse drug experience that results in any of the following outcomes:

- **Death.**
- **Life-threatening situation** — The subject was at risk of death at the time of the adverse event/experience. It does not refer to the hypothetical risk of death if the AE were more severe or were to progress.
- **Inpatient hospitalization or prolongation of existing hospitalization**.

- **Persistent or significant disability/incapacity** — Any AE having an outcome that is associated with a substantial disruption of the ability to carry out normal life functions, including the ability to work. This is not intended to include transient interruption of daily activities.
- **Congenital anomaly/birth defects —** Any structural abnormality in subject's offspring that occurs after intrauterine exposure to treatment.

Important medical events/experiences that may not result in death, be life-threatening, or require hospitalization may be considered a serious adverse event/experience when, based upon appropriate medical judgment, **they may jeopardize the subject and may require medical or surgical intervention to prevent one of the outcomes listed above,** i.e., death, a life-threatening adverse event/experience, inpatient hospitalization or prolongation of existing hospitalization, a persistent or significant disability/incapacity, or a congenital anomaly/birth defect. Examples of such medical events/experiences include allergic bronchospasm requiring intensive treatment in an emergency room or at home, blood dyscrasias or convulsions that do not result in inpatient hospitalization, or the development of drug dependency or drug abuse.

Spontaneous and elective abortions will be reported as serious adverse events.

Please note that a severe adverse event/experience is not necessarily serious, as the term severe is a measure of intensity while a serious adverse event is determined based on the aforementioned regulatory criteria.

6.3 Toxicity Management

For the purpose of medical management, all adverse events and laboratory abnormalities that occur during the study must be evaluated by the investigator. Appendix C contains "Clinical Toxicity Grades." This table is to be used in the grading of adverse events. All adverse events and laboratory abnormalities will be followed to satisfactory clinical resolution. The following guidelines should be used for study drug-related toxicity management for all subjects.

6.3.1 Grades 1-2

Subjects who develop a study drug-related Grade 1 or 2 adverse event or laboratory abnormality may continue study medications.

6.3.2 Grades 3-4

Subjects who develop a study drug-related Grade 3 or 4 adverse event or laboratory abnormality (with the exception of hyperglycemia, or Grade 3 AST/ALT elevations; (see Sections 6.3.3 and 6.3.4) should interrupt all study medications. Upon resolution of the adverse event or laboratory abnormality to within one grade level (not to exceed Grade 2) of the subject's baseline level, the subject may resume study drug dosing under the guidance of the investigator. If the subject experiences a recurrence of a study drug-related Grade 3 or 4 adverse event or laboratory abnormality after restarting study medications, the subject should interrupt all study medications again and resume study medications when the adverse event or laboratory abnormality has resolved to within one grade level (not to exceed Grade 2) of the subject's baseline.

If the subject's adverse events or laboratory abnormalities have not resolved to within one grade (not to exceed Grade 2) of his or her baseline level within eight weeks of study drug interruption, the subject should be discontinued from the study.

6.3.3 Hyperglycemia

Subjects who experience study drug-related glucose elevations of Grade 3 or 4 may continue study medications, provided that appropriate management of hyperglycemia is instituted in a timely manner. A confirmatory fasting glucose level should be obtained within two weeks after the first Grade 3 or 4 glucose elevation. Hyperglycemia may be managed with oral hypoglycemic agents or insulin as deemed clinically appropriate by the investigator.

6.3.4 Grade 3 AST/ALT Elevations

The initiation of highly active antiretroviral therapy in subjects with a history of chronic active viral hepatitis (Hepatitis C) may lead to elevations in liver function tests more frequently than in subjects without viral hepatitis. Any subject with baseline positive serology for hepatitis C (HCV) antibody who demonstrates Grade 3 elevations of liver function tests (AST, ALT) may continue study drugs in the absence of Grade 3 elevations in alkaline phosphatase or total bilirubin, only if the subject has no signs or symptoms of clinical hepatitis and does not report dark urine or clay colored stools. In such a case, clinical evaluation and/or liver function monitoring should be performed as clinically indicated. Signs of clinical hepatitis include icterus, abdominal tenderness and hepatosplenomegaly. Symptoms of clinical hepatitis include symptoms such as fever, abdominal pain, anorexia, nausea, vomiting, fatigue, malaise, and myalgias. If the subject develops signs or symptoms of clinical hepatitis, or if the AST/ALT elevates to Grade 4, study medications should be interrupted. Upon resolution of signs and symptoms and improvement of liver function test abnormalities to within one grade level (not to exceed Grade 2) of the subject's baseline level, the subject may restart study medications under the guidance of the investigator.

Subjects without a history of positive laboratory testing for HCV antibody should be evaluated for other causes of acute hepatitis, including viral serologies. Study drugs should be interrupted if an alternative etiology for the Grade 3 AST/ALT elevation is not evident.

6.3.5 Pancreatitis/Grade 3–4 Elevations of Pancreatic Amylase

A diagnosis of pancreatitis should be considered if clinical symptoms such as nausea, vomiting, or abdominal pain are present. Patients with these signs and symptoms should be appropriately evaluated for the presence of

pancreatitis through the use of diagnostic testing which may include serum amylase and lipase measurement and/or pancreatic imaging via abdominal ultrasound and/or CT scanning as clinically warranted. If a diagnosis of pancreatitis is confirmed, all drugs in the study regimen should be interrupted immediately.

If a grade 3–4 elevation of pancreatic amylase occurs during the course of the study, all drugs in the study regimen should be interrupted immediately. The subject should be evaluated for signs and symptoms of pancreatitis and additional diagnostic evaluation should be performed as clinically indicated.

If a diagnosis of pancreatitis is confirmed, appropriate therapy should be initiated. The investigator should not reintroduce study drug therapy until clinical signs and symptoms of pancreatitis have resolved and amylase values return to within normal range.

6.4 HIV-Related Clinical Events

HIV-infected subjects participating in clinical trials may develop conditions typically associated with AIDS. These conditions include certain malignancies, opportunistic infections, neurological dysfunction and some generalized constitutional symptoms. Appendix D contains a list of known expected manifestations of HIV infection. The events listed in Appendix D will be summarized as HIV-Related Events, not as Adverse Events.
The same form will be used to record Adverse Events and HIV-Related Events. HIV-Related Events that are serious in nature, as defined in Section 6.2, must be reported in the same manner as Serious Adverse Events.

6.5 Subject Management

Study participants with a single viral rebound >50 copies/mL will continue on their randomized ARV therapy. The investigator will work with the patient to

resolve possible adherence problems. Viral load will be retested after not less than 7 and not more than 30 days. If the second viral load is <50, the patient will resume the normal study visit schedule; if it is between 50 and 200, viral load will be retested weekly until either <50 or >200. If any retest viral load is >200, patients in the 3-drug HAART continuation arm will be considered to have met a study endpoint of virologic failure, and treatment will be modified at the discretion of the investigator or the treating physician. In the monotherapy arm, if any retest viral load is > 200 copies/ml, intensification with two NRTI's is allowed (either the same NRTIs as before randomization or different ones) and the subject will be considered still on randomized treatment. These patients will resume the study visit schedule starting from baseline and have viral load retested at days 15, 30, etc. After intensification, viral load results >50 will be handled as described above. Patients who have intensified will be considered to have reached the study endpoint of virologic failure if they have one viral load >50 after intensification and a consecutive viral load >200; therapy will then be modified at the discretion of the investigator or the treating physician.

"Participants who experience virologic failure, as defined above, will have HIV genotyping performed on the archived plasma sample collected at the time of the 2nd consecutive HIV RNA rebound. At the discretion of the investigator and/or treating physician, prior to treatment modification or intensification, patients may remain on their randomized ARV therapy until the HIV genotype results are available. The choice of specific agents or regimens for salvage of treatment failure will be left to the discretion of the investigator and/or treating physician, taking into account all available information including comorbid conditions, adherence issues, prior toxicities, etc."

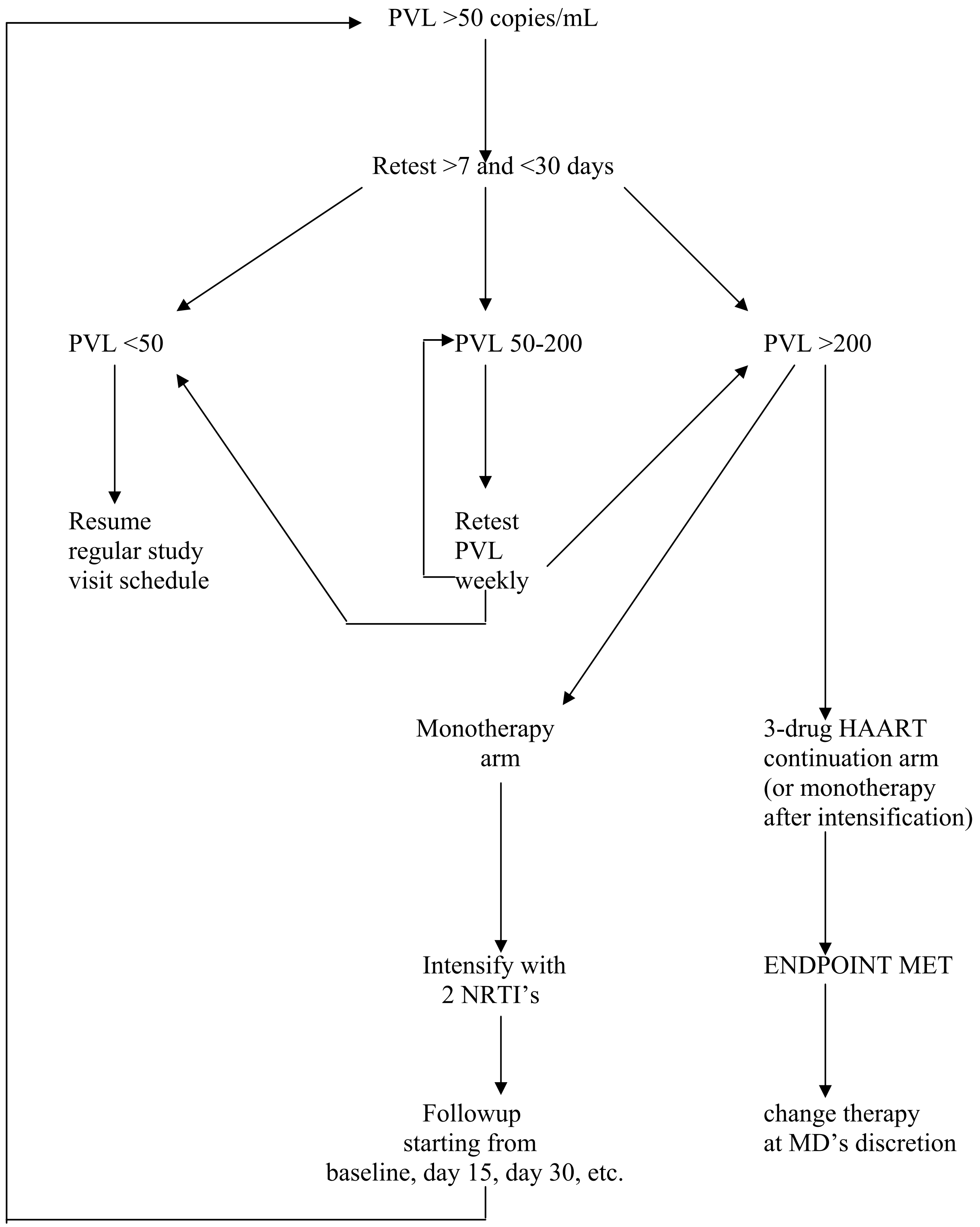
PVL >50 copies/mL
Retest >7 and <30 days
PVL <50
PVL 50-200
PVL >200
Resume regular study visit schedule
Retest PVL weekly
Monotherapy arm
3-drug HAART continuation arm (or monotherapy after intensification)
Intensify with 2 NRTI's
ENDPOINT MET
Followup starting from baseline, day 15, day 30, etc.
change therapy at MD's discretion

7.0 Statistical Considerations

7.1 Justification of Sample Size

Calculation of the sample size requirements for the current study was based on the primary objective and the primary efficacy variable of proportion of patients with virologic control at 360 days. The data in the literature have shown that at 48 weeks approximately 90% of patients virologically controlled patients on Kaletra remain virologically suppressed. In order to detect a relative risk for being virologically suppressed of 1.35 with 80% power and 5% significance level at two-tails, a total of 50 fully evaluable patients per group will be required.

7.2 Statistical Methods to Address the Study Objectives

7.2.1 Efficacy

The primary efficacy outcome variable is rate of virologic control defined as the proportion of patients with viral load count less than 200 copies / mL at 360 days. Between-group differences with respect to this variable will be assessed with the Chi-Square Statistic and the relative risk with 95% confidence intervals. Multiple logistic regression will be used to adjust the between group differences for potential confounders including patient's age and baseline characteristics. Similar analyses will be performed for the secondary efficacy variable of proportion of patients with viral load less than 50 copies/mL at 360 days. For both the <200 and <50 copies/mL outcomes, both intent-to-treat and on-treatment analyses will be performed.

The secondary efficacy variable of time to virologic rebound will be analyzed with the Kaplan Meier survival function and Breslow-Day log-rank test to compare the two groups. Cox'x proportional hazards model will be used to adjust the between group differences with respect to the rate of virologic rebound, for potential confounders including patient's age and baseline characteristic.

The Student's t-test for independent samples will be used to assess between group differences with respect to the change in viral load and CD4 cell counts between the baseline and final assessments. Multiple linear regression will be used to adjust the between group differences with respect to these outcome variables for potential confounders including patient's age and baseline characteristics.

The emergence of resistance strains will be described for those patients that experience treatment failure only. The presence of resistant mutations will be described as the percentage of patients with treatment failure that are infected with a resistant HIV mutation.

7.2.2 Safety

Safety will be assessed by the incidence of adverse events including lipid abnormalities. The relative risk will be used to assess the between group differences with respect to the incidence of adverse events. Ninety-five percent confidence intervals will be

used to assess the statistical significance and precision of the relative risk estimate. All adverse events will be described using the MedDra or WHO classification system

7.3 Level of Significance to be Used

All statistical tests will be considered as significant if the alpha level is below 5%. However, alpha levels below 10% will be considered as indicating a trend,

7.4 Criteria for Termination of the Study

For reasonable cause, the Sponsor or any regulatory agencies may terminate this study at any time.

7.5 Procedure for Handling Missing, Unused, and Spurious Data

No imputations or replacement of missing data will be performed. Observations will be censored at the time of last follow up. The last observation carried forward approach will NOT be used.

7.6 Procedures for Reporting Deviations from the Original Statistical Plan

Any deviations will be reported as amendments to the statistical analysis plan.

7.7 Criteria for Selection of Subjects to be Included in the Analyses

The primary analyses will be according to the Intent to Treat (ITT) principle that will be based on all patients enrolled in the study and have taken at least one dose of the study medications. Patients that do not adhere to the study protocol and are not compliant with the study treatment

regimen (> 80% of medications) will be excluded from the as per protocol analysis.

8.0 Direct Access to Source Data/Documents

Upon the request of the regulatory authority, IRB/IEC members, or auditors, the investigators will make all requested trial-related documents available for direct access.

9.0 Ethical Conduct of the Study

9.1 Informed Consent

It is the responsibility of the investigator to ensure that each subject is given adequate explanation of the aims, methods, anticipated benefits and potential risks of this study and voluntarily signs and dates the IRB/IEC-approved informed consent form prior to study participation. The investigator must also explain that subjects have the right to refuse to participate in the study or to withdraw at any time for any reason.

The investigator will document in the subject's medical record that informed consent was obtained prior to the performance of any study related procedure and will retain the original consent form with the study records. A copy of the signed and dated consent form should be given to the subject. Appendix G contains the elements of informed consent.

9.2 Conduct of the Study

The study will be conducted in accordance with the protocol, applicable clinical research regulations and guideline, and all applicable local regulations.

10.0 Data Handling and Record Keeping

Case Report Forms (CRFs) will be used to store information collected during this study. CRFs will be completed for each subject enrolled in this study. All case report forms will be legible and completed in ink. Any necessary corrections will be made by drawing a single line through the incorrect entry and writing in the revision, and will be initialed and dated by the investigator or his/her designee. Data will not be obliterated by blacking out, use of correction fluid, or by erasing the original entry. If the reason for the correction is not obvious, a brief explanation (e.g., transcription error) will accompany the change. All information written on the CRFs will also be reflected in the subjects' source documents.

11.0 Data Quality Assurance

In order to maintain the integrity of study data, information collected on CRFs and laboratory results will be verified by the investigator. This will be documented by the investigator´s signature.

The investigator will keep a screening/enrollment log and complete identification information on each subject to be used for the purpose of long-term follow up if needed.

12.0 Reference List

1. **Egger M, Hirschel B, Francioli P, *et al*.** Impact of new anti-retroviral combination therapies in HIV-infected patients in Switzerland: prospective, multicenter study, *Br Med J. 1997;315:1194-5.*

2. **Hogg R, Yip B, Kully C, *et al.*** Improved survival among HIV-infected patients after initiation of triple-drug antiretroviral regimens. *CMAJ 1999; 160:659-65.*

3. **Flepp M, Schiffer V, Weber R, Hirschel B.** Modern anti-HIV therapy. *Swiss Med Wkly 2001;131:207-13.*

4. **Mangum EM, Graham KK.** Lopinavir-Ritonavir: a new protease inhibitor. Pharmacotherapy 2001;21:1352-63

5. **Sham HL, Kempf DJ, Akhteruzammen M, *et al.*** ABT-378, a highly potent inhibitor of the human immunodeficiency virus protease. *Antimicrob Agents Chemother. 1998;42:3218-24.*

6. **Murphy RL, Brun S, Hicks C, et al.** ABT-378/ritonavir plus stavudine and lamivudine for the treatment of antiretroviral-naive adults with HIV-1 infection: 48-week results. *AIDS 2001;15:F1-9*

7. **Walmsley S, Bernstein B, King M, *et al.*** Lopinavir-ritonavir versus nelfinavir for the initial treatment of HIV infection. Lopinavir-ritonavir versus nelfinavir for the initial treatment of HIV infection. *N Engl J Med. 2002;346:2039-46.*

8. **Benson CA, Deeks SG, Brun SC, *et al.*** Safety and antiviral activity at 48 weeks of lopinavir/ritonavir plus nevirapine and 2 nucleoside reverse-transcriptase

inhibitors in human immunodeficiency virus type 1-infected protease inhibitor-experienced patients. *J Infect Dis. 2002;185:599-607.*

9. **Saez-Llorens X, Volari A, Deetz CO, *et al.*** Forty-eight-week evaluation of lopinavir/ritonavir, a new protease inhibitor, in human immunodeficiency virus-infected children. *Pediatr Infect Dis J. 2003;22:216-24.*

10. **Murphy R, Brun S, King M, *et al.*** Lopinavir/ritonavir (Kaletra) in antiretroviral-naïve HIV+ patients: 4 year follow-up. *42nd Interscience Conference on Antimicrobial Agents and Chemotherapy. San Diego, September 2002 [Abstract].*

11. **Cameron DW, Japour AJ, Xu Y, *et al*.** Ritonavir and saquinavir combination therapy for the treatment of HIV infection. *AIDS 1999;13:213–224.*

12. **Cohen C, Shen Y, *et al.*** Effect of nucleoside (NRTI) intensification on prevalence of morphologic abnormalities at year 5 of ritonavir (RTV) plus saquinavir (SQV) therapy in an HIV infected cohort. *9th Conference on Retroviruses and Opportunistic Infections, Seattle, USA, 2002. Poster 683-T.*

13. **Lee GA, Seneviratne T, *et al.*** Metabolic effects of lopinavir/ritonavir (Kaletra) in healthy HIV-seronegative men. *10th Conference on Retroviruses and Opportunistic Infections, Boston, USA, February 2003. Poster #748.*

14. **Pierone G Jr.,Mieras J, Kantor C, *et al.*** Initial clinical experience with Kaletra (LPV/r) monotherapy in HIV infection. *Frontiers in Drug Development for Antiretroviral Therapies (HIV DART 2002), Naples, USA, December 2002. Poster 076.*

15. **Gathe JC Jr, Washington M, Mayberry C, Piot D.** Pilot study of the safety and efficacy of Kaletra (LPV/r) as single drug HAART in HIV+ ARV naïve patients interim analysis of subjects completing at least 24 weeks of a 48 week study. *43rd. Annual ICAAC Conference, Chicago, USA, September 2003. Poster 2608.*

16. **Coté HC, Brumme ZL, Craib KJ, *et al.*** Changes in mitochondrial DNA as a marker of nucleoside toxicity in HIV-infected patients. *N Engl J Med. 2002;346:811-20*.

13.0 Appendices

Appendix A. Protocol Synopsis

Protocol Title: A Pilot, Randomized , Open-Label Study Assessing Safety, Tolerability, Efficacy of a Simplified Lopinavir/Ritonavir-Based Induction/Maintenance Therapy in HIV-Infected Subjects on their First Protease Inhibitor-Based Regimen.

Study Objectives:

Primary: The primary objective of this study is to assess the efficacy, and safety of the strategy of switching to a simplified lopinavir/ritonavir-based treatment regimen with intensification by reinitiation of 2 NRTI's if necessary, compared to the strategy of continuing on a current regimen comprising 2 NRTIs plus a ritonavir-boosted PI in patients infected with HIV-1 who are on their first PI-based anti-retroviral therapy.

Secondary:

- To evaluate the resistance profile of the simplified treatment *vs*. the control group.
- Evaluate the comparative cost of both arms at the end of the study period

Patient Population: HIV-infected male and female subjects aged ≥18 years who are on their first protease inhibitor-based treatment regimen.

Study Design: A pilot , prospective, randomized, open label, comparative, multi-center study.

Regimens: 100 subjects will receive either lopinavir/ritonavir (Kaletra™) 133.3/33.3 mg, 3 capsules BID or their usual treatment regimen.

Duration: 360 days

Route: Oral

Appendix B. Quality of Life Questionnaire

QL0730(000)/00-00-00

SYMPTOMS DISTRESS MODULE

NIAID ADULT AIDS CLINICAL TRIALS GROUP **Page 1 of 3**

Patient Number ☐☐ ☐☐☐☐ ☐ Date of Patient Visit ☐☐☐ ☐☐ ☐☐☐☐ (mmm dd yyyy)

Protocol Number ☐☐☐☐☐☐ Institution Code ☐☐☐☐☐☐

Form Week ☐☐☐ * Seq No. ☐ **Step No. ☐ Key Operator Code ☐☐☐

* Enter a "1" if this is the first of this form for this date. Designate subsequent forms on the same date with a 2, 3, etc.
**Enter the subject's current study step number. Enter '1' if the study does not have multiple steps.

FOR OFFICE USE ONLY - TEAR OFF SHEET

INSTRUCTIONS TO THE STUDY NURSE:

The SYMPTOMS DISTRESS MODULE should be given to the subject prior to the clinical exam and preferably in a quiet secluded area (e.g., exam room or other office). The subject must be able to read at the sixth-grade level at a minimum to complete the questionnaire without additional assistance.

It is important to be familiar with the content and format of the questionnaire before giving it to study participants. At the first visit, please begin by telling the subject:

> "We would like you to answer some questions about the kinds of symptoms and feelings you have been having. Your answers will help us understand the effects of the medication you are taking. We appreciate your filling out this questionnaire."

You should then briefly go over the format of the questions and how to complete them. Have the subject complete the questionnaire before vital signs, history, and physical are completed.

The questionnaire is very brief and should take no more than 5 minutes to complete. Before giving the subject the questionnaire, please fill out the header(s) and DETACH THIS PAGE.

Each question is in the same general format and contains several items. Note that the subject is always asked to make a "✓" in the box that comes the closest to how he/she has been feeling.

For data keying, if the subject did not answer a question, enter "-1."

PLEASE COMPLETE THE FOLLOWING ITEMS AFTER SUBJECT COMPLETES THE QUESTIONNAIRE OR AFTER YOU ASCERTAIN THAT THIS IS NOT POSSIBLE:

1. How was the questionnaire completed? .... ☐
   1-Self administered by the study subject
   2-Face-to-face interview that you conducted
   3-Both self-administered and interview
   4-Not completed
   9-Other, specify

   If Other, specify [30]:______________________________

   a. If you answered "4-Not completed," please indicate the reason why : ☐
      1-Subject refused
      2-Subject missed clinic visit
      3-There was not enough time
      9-Other reason, specify

   If Other, specify [30]:______________________________

09-18-98/04-22-99

QL0730(000)/00-00-00

SYMPTOMS DISTRESS MODULE

NIAID ADULT AIDS CLINICAL TRIALS GROUP

Patient Number ☐☐ ☐☐☐☐☐ ☐   Date of Patient Visit ☐☐☐ ☐☐ ☐☐☐☐ (mmm dd yyyy)

Protocol Number ☐☐☐☐☐☐   Institution Code ☐☐☐☐☐☐

Form Week ☐☐☐   * Seq. No. ☐   ** Step No. ☐   Key Operator Code ☐☐☐

INSTRUCTIONS: Please answer the following questions by placing a "✓" in the appropriate box.

A. The following questions ask about symptoms you might have had during the **past four weeks.** Please check the box that describes how much you have been bothered by **each** symptom.

(Check one.)	I DO NOT HAVE THIS SYMPTOM	I HAVE THIS SYMPTOM AND... It doesn't bother me	It bothers me a little	It bothers me	It bothers me alot
1. Fatigue or loss of energy?	☐ 0	☐ 1	☐ 2	☐ 3	☐ 4
2. Fevers, chills or sweats?	☐ 0	☐ 1	☐ 2	☐ 3	☐ 4
3. Feeling dizzy or lightheaded?	☐ 0	☐ 1	☐ 2	☐ 3	☐ 4
4. Pain, numbness or tingling in the hands or feet?	☐ 0	☐ 1	☐ 2	☐ 3	☐ 4
5. Trouble remembering?	☐ 0	☐ 1	☐ 2	☐ 3	☐ 4
6. Nausea or vomiting?	☐ 0	☐ 1	☐ 2	☐ 3	☐ 4
7. Diarrhea or loose bowel movements?	☐ 0	☐ 1	☐ 2	☐ 3	☐ 4
8. Felt sad, down or depressed?	☐ 0	☐ 1	☐ 2	☐ 3	☐ 4
9. Felt nervous or anxious?	☐ 0	☐ 1	☐ 2	☐ 3	☐ 4
10. Difficulty falling or staying asleep?	☐ 0	☐ 1	☐ 2	☐ 3	☐ 4
11. Skin problems, such as rash, dryness or itching?	☐ 0	☐ 1	☐ 2	☐ 3	☐ 4

09-18-98/04-22-99

4696

QL0730(000)/00-00-00

SYMPTOMS DISTRESS MODULE **Page 3 of 3**

Pt. No. ☐☐ ☐☐☐☐ ☐ * Seq. No. ☐ ** Step No. ☐ Date ☐☐☐ (mmm) ☐☐ (dd) ☐☐☐☐ (yyyy)

(Check one.)	I DO NOT HAVE THIS SYMPTOM	I HAVE THIS SYMPTOM AND...			
		It doesn't bother me	It bothers me a little	It bothers me	It bothers me alot
12. Cough or trouble catching your breath?	☐ 0	☐ 1	☐ 2	☐ 3	☐ 4
13. Headache?	☐ 0	☐ 1	☐ 2	☐ 3	☐ 4
14. Loss of appetite or a change in the taste of food?	☐ 0	☐ 1	☐ 2	☐ 3	☐ 4
15. Bloating, pain or gas in your stomach?	☐ 0	☐ 1	☐ 2	☐ 3	☐ 4
16. Muscle aches or joint pain?	☐ 0	☐ 1	☐ 2	☐ 3	☐ 4
17. Problems with having sex, such as loss of interest or lack of satisfaction?	☐ 0	☐ 1	☐ 2	☐ 3	☐ 4
18. Changes in the way your body looks such as fat deposits or weight gain?	☐ 0	☐ 1	☐ 2	☐ 3	☐ 4
19. Problems with weight loss or wasting?	☐ 0	☐ 1	☐ 2	☐ 3	☐ 4
20. Hair loss or changes in the way your hair looks?	☐ 0	☐ 1	☐ 2	☐ 3	☐ 4

Thank you very much for completing this questionnaire.

Language: English [E]

09-18-98/04-22-99

Date Form Keyed (DO NOT KEY): ____ / ____ / ____

Appendix C. Clinical Toxicity

	GRADE 1 TOXICITY	GRADE 2 TOXICITY	GRADE 3 TOXICITY	GRADE 4 TOXICITY
**HEMATOLOGY**				
HEMOGLOBIN<br><br>HEMOGLOBIN<br>(S.I. Units)	9.6–10.5 g/dl<br><br>96–105 g/L	8.0–9.5 g/dl<br><br>80–95 g/L	6.5–7.9 g/dl<br><br>65–79 g/L	< 6.5 g/dl<br><br>< 65 g/L
ABSOLUTE NEUTROPHIL COUNT<br><br>ABSOLUTE NEUTROPHIL COUNT<br>(S.I. Units)	1000–1500/mm^3<br><br>1000–1500 GI/L	750–999/mm^3<br><br>750–999 GI/L	500–749/mm^3<br><br>500–749 GI/L	<500/mm^3<br><br><500 GI/L
PLATELETS<br><br>PLATELETS<br>(S.I. Units)	75,000–99,000/mm^3<br><br>75,000–99,000 GI/L	50,000–74,999/mm^3<br><br>50,000–74,999 GI/L	20,000–49,999/mm^3<br><br>20,000-49,999 GI/L	<20,000/mm^3or diffuse petechiae<br><20,000 GI/L or diffuse petechiae
PT	.01–1.25 x upper limit of normal	1.26–1.5 x upper limit of normal	1.51–3.0 x upper limit of normal	3 x upper limit of normal
APTT	1.20–1.66 x upper limit of normal	0.67–2.35 x upper limit of normal	.36–3 x upper limit of normal	>3 x upper limit of normal
FIBRINOGEN	0.99–0.75 x lower limit of normal	0.74–0.50 x lower limit of normal	0.49–0.25 x lower limit of normal	<0.25 x lower limit of normal
FIBRIN SPLIT PRODUCT	20–40 µg/ml	41–50 µg/ml	51–60 µg/ml	>60 µg/ml
METHEMOGLOBIN	5.0–9.9%	10.0–14.9%	15.0–20.0%	>20%
**CHEMISTRIES**				
HYPONATREMIA<br><br>HYPONATREMIA<br>(S.I. Units)	130-134 mEq/L<br><br>130–134 mmol/L	123–129 mEq/L<br><br>123–129 mmol/L	116–122 mEq/L<br><br>116–122 mmol/L	115 mEq/L and less or mental status changes or seizures<br>15 mmol/L and less or mental status changes or seizures
HYPERNATREMIA<br><br>HYPERNATREMIA<br>(S.I. Units)	146–150 mEq/L<br><br>146–150 mmol/L	151–157 mEq/L<br><br>151–157 mmol/L	158–165 mEq/L<br><br>158–165 mmol/L	165 mEq/L or mental status changes/seizures<br>165 mmol/L or mental status changes/seizures
HYPOCALCEMIA<br>(ionized calcium)	3.0–3.4 mg/dL	2.5–2.9 mg/dL replacement Rx req.	2.0–2.4 mg/dL or intensive replacement Rx Req. or	<2 mg/dL or paresis or ileus or life–threatening

			hospitalization	arrhythmia
HYPERCALCEMIA (ionized calcium)	5.6–6.0 mg/dL	6.1–6.5 mg/dL	6.6–7.0 mg/dL	>7.0 mg/dL or paresis or ileus or life–threatening arrhythmia
TRIGLYCERIDES<br>TRIGLYCERIDES (S.I. Units)	– — — — — — — — — — – –<br>– — — — — — — — — — – –	400–750 mg/dL<br>4.52–8.47 mmol/L	751–1200 mg/dL<br>8.48–13.55 mmol/L	>1200 mg/dL<br>>13.55 mmol/L
CHOLESTEROL<br>CHOLESTEROL (S.I. Units)	200–239 mg/dL<br>5.16–6.19 mmol/L	240–300 mg/dL<br>>6.19–7.77 mmol/L	301–400 mg/dL<br>>7.77–10.35 mmol/L	>400 mg/dL<br>>10.35 mmol/L
HYPOGLYCEMIA<br>HYPOGLYCEMIA (S.I. Units)	55–66 mg/dL<br>3.03–3.69 mmol/L	40–54 mg/dL<br>2.20–3.02 mmol/L	30–39 mg/dL<br>1.64–2.19 mmol/L	30 mg/dL or mental status changes or coma<br>1.64 mmol/L or mental status changes or coma
HYPERGLYCEMIA<br>HYPERGLYCEMIA (S.I. Units)	116–160 mg/dL<br>6.42–8.91 mmol/L	161–250 mg/dL<br>8.92–13.90 mmol/L	251–500 mg/dL<br>13.91–27.79 mmol/L	500 mg/dL or ketoacidosis or seizures<br>>27.79 mmol/L or ketoacidosis or seizures
HYPERURICEMIA<br>HYPERURICEMIA (S.I. Units)	7.5–9.9 mg/dL<br>441–591 µmol/L	10.0–12.0 mg/dL<br>592–716 µmol/L	12.1–15.0 mg/dL<br>717–895 µmol/L	>15.0 mg/dL<br>>895 µmol/L
HYPOCALCEMIA corrected for albumin	8.4–7.8 mg/dL	7.7–7.0 mg/dL	6.9–6.1 mg/dL	<6.1 mg/dL or life–threatening arrhythmia or tetany
HYPERCALCEMIA corrected for albumin	10.6–11.5 mg/dL	11.6–12.5 mg/dL	12.6–13.5 mg/dL	>13.5 mg/dL or life–threatening arrhythmia or tetany
HYPOMAGNESEMIA	1.4–1.2 mEq/L	1.1–1.0 mEq/L or replacement of Rx req.	0.9–0.6 mEq/L or intensive Rx req. hospitalization	<0.6 mEq/L or life–threatening arrhythmia
HYPOMAGNESEMIA (S.I.Units)	0.70–0.56 mmol/L	0.55–0.44 mmol/L or replacement of Rx req.	0.43–0.30 mmol/L or intensive Rx req. hospitalization	<0.30 mmol/L or life-threatening arrhythmia
HYPOPHOSPHATEMIA<br>HYPOPHOSPHATEMIA (S.I.Units)	2.0-2.4 mg/dL<br>0.63–0.79 mmol/L	1.5–1.9 mg/dL or replacement Rx req.<br>0.47–0.62 mmol/L or replacement Rx req.	1.0–1.4 mg/dL intensive Rx req. hospitalization<br>0.31–0.46 mmol/L intensive Rx req. hospitalization	<1.0 mg/dL life–threatening arrhythmia or CHF<br><0.31 mmol/L life–threatening arrhythmia or CHF
HYPER–BILIRUBINEMIA	1.1–1.5 x upper limit of normal	1.6–2.9 x upper limit or normal	3–5 x upper limit of normal	>5 x upper limit of normal
HYPER–	22–31 µmol/L	>31–62 µmol/L	>62–103 µmol/L	>103 µmol/L

BILIRUBINEMIA (S.I.Units)	(Grade 1)	(Grade 2)	(Grade 3)	(Grade 4)
HYPOKALEMIA<br>HYPOKALEMIA (S.I.Units)	3.0–3.4 mEq/L<br>3.0–3.4 mmol/L	2.5–2.9 mEq/L<br>2.5–2.9 mmol/L	2.0–2.4 mEq/L<br>2.0–2.4 mmol/L	<2.0 mEq/L<br><2.0 mmol/L
HYPERKALEMIA	5.6–6.0 mEq/L	6.1–6.5 mEq/L	6.6–7.0 mEq/L	>7.0 mEq/L
HYPERKALEMIA (S.I.Units)	5.6–6.0 mmol/L (Grade 1)	6.1–6.5 mmol/L (Grade 2)	6.6–7.0 mmol/L (Grade 3)	>7.0 mmol/L (Grade 4)
BUN	1.25–2.5 x upper limit of normal	2.6–5.0 x upper limit of normal	5.1–10.0 x upper limit of normal	>10 x normal
CREATININE	1.1–1.5 x upper limit of normal	1.6–3.0 x upper limit of normal	3.1–6.0 x upper limit of normal	>6.0 x upper limit of normal or requires dialysis
CPK (CK) (not related to exercise)	1.1–2.0 x upper limit of normal	2.1–4.0 x upper limit of normal	4.1–6.0 x upper limit of normal	>6.0 x upper limit of normal
**ENZYMES**				
AST/SGOT	1.25–2.5 x upper limit of normal	2.6–5 x upper limit of normal	5.1–10 x upper limit of normal	>10 x upper limit of normal
ALT/SGPT	1.25–2.5 x upper limit of normal	2.6–5 x upper limit of normal	5.1–10 x upper limit of normal	>10 x upper limit of norm
GGT	1.25–2.5 x upper limit of normal	2.6–5 x upper limit of normal	5.1–10 x upper limit of normal	>10 x upper limit of normal
ALKALINE PHOSPHATASE	1.25–2.5 x upper limit of normal	2.6–5 x upper limit of normal	5.1–10 x upper limit of normal	>10 x upper limit of normal
AMYLASE (pancreatic)	1.1–1.3 x upper limit of normal	1.4–2.0 x upper limit of normal	2.1–5.0 x upper limit of normal or mild to moderate clinical pancreatitis	5.1 x upper limit of normal or severe clinical pancreatitis
**URINALYSIS**				
PROTEINURIA	1+, 30–100 mg/dL	2+, >100–300 mg/dL	3+, >300 mg/dL	nephrotic syndrome
HEMATURIA	microscopic only, <10 RBCs	gross, no clots, 10–100 RBCs	gross, clots, > 101 RBCs	obstructive or Rx req.
**CARDIAC**				
CARDIAC RHYTHM	- -- - - - - - -- - - -	asymptomatic, transient signs, no Rx required	recurrent/persistent, no Rx required	unstable dysrhythmia requires treatment or hospitalization
HYPERTENSION	transient inc. >20 mm, no Rx	recurrent, chronic >20 mm, Rx req.	requires outpt. acute Rx	hospitalization
HYPOTENSION	transient orthostatic hypotension, no Rx	symptoms correctable with oral fluid Rx	requires IV fluids, no hospitalization	requires hospitalization

PERICARDITIS	minimal effusion	mild/mod asymptomatic effusion no Rx	symptomatic effusion pain ECG changes	tamponade percardiocentesis or surgery required
HEMORRHAGE, BLOOD LOSS	microscopic occult	mild/no transfusion	gross blood loss, 1–2 units transfused	massive blood loss, >3 units transfused
**RESPIRATORY**				
COUGH	transient-no Rx	treatment associated cough, local non-narcotic Rx	treatment associated cough, narcotic Rx required	uncontrolled
SHORTNESS OF BREATH	mild, does not interfere with routine activities	moderate, interferes with routine activities req. intermittent Rx	moderately debilitating requiring nasal oxygen	severe, requiring ventilatory assistance
BRONCHOSPASM ACUTE	transient, no Rx, 70%–80% of peak flow	req. Rx normalize w/bronchodilator FEV at 50% of peak flow	no normalization w/bronchodilator FEV at 50% of peak flow, retraction	cyanosis, FEV <25% of peak flow intubated
**GASTROINTESTINAL**				
STOMATITIS	mild discomfort, no limits on activity	some limits on eating, talking	eating, talking very limited	unable to drink fluids; req. IV fluids
NAUSEA	transient, mild discomfort, maintain reasonable intake	mod. discomfort, sign dec of intake, some limit of activity or decreased intake <3 days	severe discomfort, no significant food intake activities limited or minimal intake˛3days	minimal fluid intake or hospitalization required
VOMITING	transient emesis, 2–3 per day or lasting <1 week	moderate emesis 4–5 per day or lasting 1week	vomiting all food/fluids in 24 hours, orthostatic hypotension or IV fluid, Rx required.	hypotensive shock hospitalization IV fluid therapy
CONSTIPATION	mild	moderate, Rx required	severe, Rx required, vomiting	distention with vomiting
DIARRHEA	mild or transient, 3–4 loose stools/day or mild diarrhea lasting <1 week	moderate or persistent; 5–7 loose stools/day or diarrhea lasting˛1 week	bloody diarrhea; or orthostatic hypotension or >7 loose stools/day or IV Rx required	hypotensive shock or hospitalization required
ABDOMINAL PAIN	mild, occasional transient	moderate, transient	severe or requiring analgesic	severe with guarding peritoneal signs
**NEURO/NEUROMUSCULAR**				
NEURO CEREBELLAR	slight incoordination Dyskiadokinesis	intention tremor, dysmetria, slurred speech, nystagmus	locomotor ataxia	incapacitated
MOOD	mild anxiety or depression	mod. anxiety or depression, therapy required	severe anxiety or depression or manic; (needs assistance)	acute psychosis; incapacitated req. hospitalization

NEUROCONTROL	confusion/agitation	mod. confusion/ agitation; some severe, min. Rx	sev. confusion/ agitation	toxic psychosis; hospitalization
MUSCLE STRENGTH	subjective weakness, no objective symptoms/signs	mild objective no dec in function	objective weakness; function limited	paralysis
PAINFUL NEUROPATHY	mild discomfort; no therapy required	moderate discomfort persisting for > 72 hours; analgesic required	severe discomfort, marked antalgic gait, narcotic analgesic required, with symptomatic improvement	incapacitating, intolerable discomfort. Not improved or unable to walk despite narcotic analgesics
MYOSITIS	minimal findings	Patients must have some measures of myositis (positive EMG or muscle biopsy) and one of the following:	Patients must have some measures of myositis (positive EMG or muscle biopsy) and one of the following:	Patients must have some measures of myositis (positive EMG or muscle biopsy) and one of the following:
		1) mild myalgias, >6 weeks requiring nonsteroidal anti-inflammatory agents	1) moderate myalgias or muscle tenderness, >6 weeks requiring non-steroidal anti-inflammatory agents	1) severe muscle pain (myalgias) not related to exercise requiring narcotics
		2) difficulty climbing stairs or rising from a sitting position but able to ambulate without assistance	2) requires some assistance with ambulation or general activities	2) muscle weakness resulting in inability to ambulate, requiring special care and assistance with mobilization
				3) acute rhabdomyolysis with muscle necrosis and edema, moderate to severe muscle weakness with inability to ambulate or mobilize self without assistance
				4) acute rhabdomyolysis associated with electrolyte imbalance in renal failure

OTHER PARAMETERS

FEVER oral, w/o infection, >12 hrs.	37.7–38.5C or 100.0–101.5F	38.6–39.5C or 101.6–102.9F	39.6–40.5C or 103–105F	>40.5C >105F
HEADACHE	mild, no Rx therapy	transient, mod; non-narcotic Rx req.	severe, responds to initial narcotic	intractable, req. repeated narcotic

			therapy	therapy
FATIGUE	<25% decrease in daily activities	normal activity decrease 25–50%	normal activity decrease >50%, can't work	unable to care for self
ALLERGIC REACTION	pruritus w/o rash	localized urticaria angioedema	generalized urticaria angioedema	anaphylaxis
LOCAL REACTION	tenderness or erythema	induration <10 cm or phlebitis or inflammation	induration >10 cm or ulceration	necrosis
MUCOCUTANEOUS	erythema, pruritus	diffuse maculopapular rash dry desquamation	vesiculation, moist desquamation ulceration	exfoliative dermatitis, mucous membrane involvement suspected, Stevens Johnson or erythema multiforme, necrosis requiring surgery

Appendix D. List of Known Expected Manifestations of HIV Infection

1. Candidiasis:

– *Bronchi
– *Esophagus
– *Lungs
– Oropharyngeal (Thrush)
– *Trachea
– Vulvovaginal (Persistent, Frequent, or Poorly Responsive to Therapy)
– Other Candidiasis

2. Cytomegalovirus:

– *Retinitis
– *Cytomegalovirus Disease (other than liver, spleen or nodes)

3. Herpes Simplex Virus

– *Bronchitis
– *Esophagitis
– *Pneumonitis
– *Chronic Ulcer(s) (> 1 month in duration)
– Other Sites

4. Lymphoma (HIV-Related)

– *Burkitt's
– *Immunoblastic
– *Primary of brain

5. Mycobacterial Disease:

– *Mycobacterium avium - intracellulare
– *Mycobacterium kansasii
– *Mycobacterium tuberculosis
    *Other Mycobacteriosis

* AIDS-defining event as described by CDC Surveillance Case Definition of 1993

Appendix D List of Known Expected Manifestations of HIV Infection (Continued)

6. Other

- *Cervical cancer, invasive
- Cervical cancer, *in situ*
- Cervical dysplasia
- *Coccidioidomycosis
- *Cryptococcosis
- *Cryptosporidiosis
- *Encephalopathy HIV-related
- Hairy leukoplakia, oral
- Herpes zoster
- *Histoplasmosis
- *Isosporiasis
- *Kaposi's sarcoma
- Listeriosis
- **Pneumocystis carinii* pneumonia
- *Pneumonia, recurrent
- *Progressive multifocal leukoencephalopathy
- *Salmonellosis
- *Septicemia, recurrent
- *Toxoplasmosis of the brain
- *Wasting syndrome, HIV-related

* AIDS-defining event as described by CDC Surveillance Case Definition of 1993

Appendix E Documents Required Prior to Initiation of the Study

Prior to the beginning of the clinical study, the investigators will be asked to provide the following documentation.

- An original Investigator-signed Protocol Agreement page.
- A current curriculum vitae for the investigator. If subinvestigators will participate in the study, a curriculum vitae is required for each additional individual.
- A copy of the signed and dated approval letter from the Institutional Review Board (IRB)/Independent Ethics Committee (IEC), with regard to protocol, informed consent, and any advertisement(s).
- A list of IRB/IEC committee members, including their occupations and institutional affiliations.
- An approved copy of the IRB/IEC informed consent document to be used in this study.
- A list of normal reference ranges and values for all laboratory tests specified by the protocol for all laboratories utilized.
- A current copy of the laboratory(ies) certification(s) or the certification number(s), the name of the certifying authority, the period of certification, and the Laboratory Director's curriculum vitae.

Appendix F Responsibilities of the Clinical Investigator

a) To secure prior approval of the study by an appropriate Institutional Review Board/Independent Ethics Committee which conforms to ICH guidelines.

b) To obtain valid written informed consent from each person who participates in the study.

c) To prepare and maintain adequate case histories of all persons entered into the study, including case report forms, hospital records, laboratory results, etc., and to maintain these data for the time required by their institution.

d) To identify all subinvestigators who will also supervise drug administration.

e) To report adverse effects to the principal investigator promptly. In the event of a serious or unexpected adverse event, to notify the principal investigator immediately by telephone.

Appendix G Elements of Informed Consent

A signed consent must be obtained prior to any study-specific activities and must include the following items:

a. A statement that the study involves research, and explanation of the purpose of the research and the expected duration of the patient's participation, a description of the procedures to be followed, and identification of any procedures that are experimental and/or invasive.
b. A description of the study, including trial treatments and the probability for random assignment to treatment.
c. A description of any reasonably foreseeable risks, inconveniences or discomforts to the patients and, if applicable, to an embryo, fetus or nursing infant.
d. A description of the subject's responsibilities.
e. A description of any benefits to the patient or to others which may reasonably be expected from the research. If the patient is to be compensated for participating in the study, the consent form must describe what the compensation consists of (to assure neither coercion nor undue influence).
f. A disclosure of appropriate alternative procedures or courses of treatment, if any, that might be advantageous to the patient, and the potential risks and benefits.
g. A statement that the investigator and the Institutional Review Board (IRB)/Independent Ethics Committee (IEC), will be granted direct access to the subject's original medical records for verification of clinical trial procedures and/or data, without violating the confidentiality of the subject, to the extent permitted by the applicable laws and regulations and that, by signing a written informed consent form, the subject or the subject's legally acceptable representative is authorizing such access.

   A statement that records identifying the subject will be kept confidential and, to the extent permitted by the applicable laws and/or regulations, will not be made publicly available. If the results of the trial are published, the subject's identity will remain confidential.
h. An explanation of whom to contact for answers to pertinent questions about the research and research patients' rights, and whom to contact in the event of a research-related injury to the patient. (NOTE: It is preferable to identify as the contact some person other than the investigator. The guidance of the IRB/IEC may be required.)

i. A statement that participation is voluntary, that refusal to participate will involve no penalty or loss of benefits to which the patient is otherwise entitled, and that the patient may discontinue participation at any time without penalty or loss of benefits to which the patient is otherwise entitled.
j. A statement that a signed and dated copy of the consent form will be given to the patient.
k. A statement of the agreement to participate (e.g., "I agree to participate ...").
l. A place for signature and date of signature for the research patient (or legally authorized representative) and for the person who explained the nature of the study to the patient (investigator or investigator's representative).
m. *A statement that the particular treatment or procedures may involve risks to the patient (or to the embryo, fetus or nursing infant, if the patient is or may become pregnant) that are currently unforeseeable.
n. A statement of anticipated circumstances or reasons under which the patient's participation may be terminated by the investigator without regard to patient's consent.
o. A statement of any additional costs to the patient that may result from participation in the research.
p. A statement regarding the consequences of a patient's decision to withdraw from the research and procedures for orderly termination of participation by the patient.
q. A statement that significant new findings developed during the course of the research which may relate to the patient's willingness to continue participation will be provided to the patient (or the patient's legally acceptable representative) in a timely manner.
r. A statement of the approximate number of patients involved in the study.

Appendix H Participating Investigators

Dr Julio Montaner, MD, FRCPC, FCCP
Professor of Medicine and
Chair in AIDS Research
University of British Columbia
1081 Burrard St., Room 667
Vancouver, BC
V6Z - 1Y6
Tel: 604-806-8036
Fax: 604-806-8527

Dr Pedro Cahn, MD
Fundación Huésped
Address of PI: Pje. Peluffo 3932 - (1202) - Ciudad de Buenos Aires - Argentina
Telephone number of PI: +54 11 4981 1855 / 7777
Fax number of PI:+54 11 4982 4024
e-mail address of PI:**pcahn@huesped.org.ar**

Dr Isabel Cassetti, MD
Helios Salud
Address of PI: Perú 1511/1515 - (1141) - Ciudad de Buenos Aires - Argentina.
Telephone number of PI : +54 11 4300 0515
Fax number of PI:+54 11 4300 5021
e-mail address of PI: **icassetti@funcei.org.ar**

Dr Juan Sierra-Madero
Instituto Nacional de Ciencias Medicas y Nutrición "Salvador Zubirán"
Address of PI: Vasco de Quiroga Num 15 Col Sección XVI, Delegación Tlalpan CP 14 000 México DF, México
Telephone number of PI: + 55 13 33 65
Fax number of PI: + 55 13 81 06
e-mail address of PI: **jsierra@quetzal.innsz.m**

APPENDIX I

Optional Pharmacokinetic Substudy

A pilot, randomized, open-label study assessing safety, tolerability, efficacy of a simplified lopinavir/ritonavir-based induction/maintenance therapy in HIV-infected subjects on their first protease-inhibitor-based regimen

Introduction

Approximately 100 HIV-infected patients enrolled at sites in Canada, Argentina, and Mexico will be randomized to either continue on their first protease-inhibitor (PI)-based antiretroviral regimen or switch to a maintenance regimen of lopinavir/ritonavir (LPV/r) (400mg/100 mg twice daily) alone ("Kaletra monotherapy"). Preliminary data supporting the efficacy of Kaletra monotherapy have recently been presented, both for antiretroviral naïve patients [1] and for those already suppressed on triple-drug LPV/r-based therapy [2].

Antiviral efficacy of PI-based regimens has been linked to plasma pharmacokinetics (PK), particularly trough levels [3,4]. While LPV/r produces relatively robust plasma levels generally well above the EC_{50} of wild-type virus [5], considerable intra- and inter-patient variability in LPV plasma levels have been observed [6,7]. Presence or absence of concomitant nucleosides are not expected to affect LPV levels, with the possible exception of tenofovir; however, in the context of LPV/r monotherapy, plasma LPV levels may be particularly critical. The results of suboptimal levels (i.e. virologic failure and the emergence of resistance) may be more likely to occur in the absence of concomitant nucleosides as backup. Of note, in the naïve study presented in Bangkok [1], LPV/r dose was adjusted upwards from the standard 400mg/100mg twice daily dose to 533mg/133mg twice daily, based on patient body weight.

Therefore, it would be of interest to see whether trough PI levels can be correlated with virologic efficacy in this study, particularly for the Kaletra monotherapy arm. While this component of the study will be optional, it will be encouraged for all patients. Separate informed consent will be sought for the PK substudy at the time patients consent to participate in the main study. For patients in both arms who consent to this procedure, an additional pre-dose trough plasma sample will be drawn at the study visits at baseline and days 15, 30, 90, 180, and 360. Plasma will be stored frozen and shipped to the laboratory of the British Columbia Centre for Excellence in HIV/AIDS in Vancouver, B.C. for measurement of PI levels.

Objectives

1. To determine whether virologic efficacy in Kaletra monotherapy and other PI-based regimens is associated with trough plasma PI levels.
2. To determine whether trough LPV levels differ between patients who are taking Kaletra with or without concomitant nucleosides.
3. To determine whether suboptimal LPV trough levels are more likely to be associated with virologic failure in patients taking Kaletra monotherapy than in those taking 2 nucleosides plus Kaletra.

Substudy procedures

At the study visits at baseline and days 15, 30, 90, 180 and 360, consenting patients will be requested to attend the clinic before their morning dose of PI medication. The time of the previous PI dose will be recorded and an additional 3-5 mL blood sample will be collected in an EDTA tube along with the usual study blood tests. Blood samples will be stored at 4°C until processing. Plasma will be separated by centrifugation within 24

hours of collection and stored at -70 ºC. Frozen plasma samples will be shipped to laboratory of the British Columbia Centre for Excellence in HIV/AIDS in Vancouver, B.C. where PI levels will be analyzed by a validated assay using HPLC coupled with tandem mass spectrometry.

Statistics

Consent to take part in the PK substudy will be sought from all participants in the main study; therefore, up to 100 patients (50 in each arm) will participate in the PK substudy. Using Student's t-test, LPV trough levels will be compared between patients randomized to Kaletra monotherapy, and those randomized to continue PI-based triple therapy who are taking Kaletra as their PI. In addition, trough PI levels will be entered as a covariate into the univariate and multivariate analysis for the efficacy endpoint of the main study (as described in section 7.2.1 of the study protocol).

References

1. Gathe JC Jr, Washington MY, Mayberry C, Piot D, Nemcek J. IMANI-1 TC3WP Single drug HAART – proof of concept study. Pilot study of the safety and efficacy of Kaletra (LPV/r) as single drug HAART in HIV+ ARV-naïve patients – interim analysis of subjects completing final 48 week data. XV International AIDS Conference, Bangkok, Thailand, July 11-16, 2004. Abstract MoOrB1057.
2. Arribas JR, Pulido F, Lorenzo A, *et al.* Simplification to lopinavir/r single-drug HAART: 24 weeks results of a randomized, open label, pilot clinical trial (OK Study). XV International AIDS Conference, Bangkok, Thailand, July 11-16, 2004. Abstract TuPeB4486.

3. Burger DM, Hoetelmans RM, Hugen PW, *et al.* Low plasma concentrations of indinavir are related to virological treatment failure in HIV-1-infected patients on indinavir-containing triple therapy. *Antivir Ther* 1998;3:215-20.
4. Burger DM, Hugen PWH, Aarnoutse RE, *et al.* on behalf of the ATHENA Study Group. Treatment failure of Nelfinavir-containing triple therapy can largely be explained by low Nelfinavir plasma concentrations. *Ther Drug Monit* 2003;25:73–80.
5. Kaletra™ Product Information. Abbott Laboratories, North Chicago, IL; October 2000.
6. Alexander CA, Montaner JS, Asselin JJ, *et al.* Dose changes, intrapatient variability, and exposure to lopinavir – implications for the use of therapeutic drug monitoring in the clinical setting. XV International AIDS Conference, Bangkok, Thailand, July 11-16, 2004. Abstract TuPeB4642.
7. Burger DM, LaPorte CJL, Schippers EF, *et al.* Pharmacokinetics of once-daily (QD) lopinavir/ritonavir (LPV/r) as part of a regimen also containing two nucleosides administered QD: the influence of dose modifications. XV International AIDS Conference, Bangkok, Thailand, July 11-16, 2004. Abstract TuPeB4607.
